# SNX20AR/MiRNA-301a-3p/SNX20 Axis Associated With Cell Proliferation and Immune Infiltration in Lung Adenocarcinoma

**DOI:** 10.3389/fmolb.2021.744363

**Published:** 2021-09-17

**Authors:** Yixiao Yuan, Xiulin Jiang, Lin Tang, Juan Wang, Qianqian Liu, Xiaolan Zou, Lincan Duan

**Affiliations:** ^1^ Department of Graduate School of Kunming Medical University, Kunming, China; ^2^ Department of Thoracic Surgery, The Third Affiliated Hospital of Kunming Medical University, Kunming, China; ^3^ Kunming College of Life Science, University of Chinese Academy of Sciences, Beijing, China

**Keywords:** lncRNA, miRNA-301a-3p, SNX20, LUAD, immune infiltration, cell proliferation

## Abstract

Lung cancer is the most common tumor with severe morbidity and high mortality. Increasing evidence has demonstrated that SNX20 plays crucial roles in the progression of human cancer. However, the functions and mechanism of SNX20 in LUAD are still barely known. Here, we employ the TCGA, GEO and CCLE databases to examine the expression of SNX20 in human varies cancer, the results shown that SNX20 is down-regulated in lung Adenocarcinoma, SNX20 level was significantly positive correlated with poor prognosis and lung cancer immune cell infiltration. We found that over-expression of SNX20 significantly restrain NSCLC cell proliferation and migration. Subsequently, we discover a network regulating SNX20 in LUAD, further study found that the decreased of the SNX20 likely caused by DNA hypermethylation. Furthermore, we identified that SNX20AR/miRNA-301a-3p mediated decreased of SNX20 correlated with lung cancer progression and cancer immune infiltration in LUAD. Our findings suggested that ncRNAs play a crucial role in the regulatory network of SNX20. Collectively, our findings demonstrate the suppressor roles of the SNX20AR/miRNA-301a-3p/SNX20 axis in Lung Adenocarcinoma, represent that SNX20 have the potential of as an effective therapeutic target in future.

## Introduction

Lung cancer is the leading cause of cancer-related death in the world (Sung et al., 2021). Lung adenocarcinoma is the most common molecular subtype of NSCLC cancer, and LUAD accounts for almost 50 percent of lung cancers. Although there are many treatments for lung cancer, the incidence rate and mortality rate of LUAD patients remains very high (Martin and Leighl, 2017). Therefore, it’s extremely urgent to discerned accurate and sensitive immune-related biomarkers and elucidate the molecular mechanisms participate in LUAD progression.

SNX20, as a member of sorting nexins proteins family, play crucial role in the functional organization (Kurten et al., 1996; Carlton et al., 2004). Previous studies demonstrated that sorting nexins was divided into different groups according to the special functional domains, mainly ranging from BAR, PDZ, FERM-like, RGS to SH3 domains (Zheng, 2001; Wassmer et al., 2009; van Weering et al., 2012). In humans, the SNX sub-group mainly including the sorting nexin-20, sorting nexin-21 and the SNX-PXB proteins (Zeng et al., 2002). These proteins has a C-terminal PX-associated B (PXB) domain which is essential for protein interactions (Clairfeuille et al., 2015). It has been shown that SNX20 able to regulation the endosomal trafficking and endothelial cell adhesion (Schaff et al., 2008). Finally, the SNX20 gene locus has recently been correlated to inflammatory bowel disease (Brant et al., 2017). Until now, there was no study focused on the function of SNX20 in LUAD progression. In this study, we aimed to investigate the role of SNX20 in Lung adenocarcinoma progression and tumor-infiltrating lymphocytes.

In this work, we find that SNX20 was significantly decreased in LUAD and it’s low expression was correlated with poor prognosis, pathological stage and lymph node metastasis. GSEA analysis suggested that high SNX20 expression was mainly enriched with the immune-related signaling pathways, such as JAK STAT signaling pathway, T cell receptor signaling pathway and Toll like receptor signaling pathway. Additionally, SNX20 was positive correlated with the different immune infiltrations and immune check point related gene expression. We show that elevated the SNX20 expression was significantly inhibits the cell proliferation and migration of NSCLC cells. Subsequently, in this study, we identified a lncRNA, termed as SNX20AR (SNX20 associated lncRNA: ENSG00000258168) was high expressed in NSCLC cancerous tissues and predicts poor prognosis. We identified that SNX20AR/miRNA-301a-3p mediated decreased of SNX20 correlated with lung cancer progression and cancer immune infiltration in LUAD. Our findings suggested that ncRNAs play a crucial role in the regulatory network of SNX20. Collectively, our findings demonstrate the suppressor roles of the SNX20AR/miRNA-301a-3p/SNX20 axis in LUAD, represent that SNX20 have the potential of as an effective therapeutic target in future.

## Materials and Methods

### The Expression, Prognosis, Clinical Information and Immune Infiltration Analysis

We mainly using the following databases to analysis the expression, prognosis, clinical information and Immune infiltration of SNX20 in cancers. The detail information of databases used in this study are as follows: Oncomine database (https://www.oncomine.org/) (Rhodes et al., 2004), TIMER (http://timer.cistrome.org/) (Li et al., 2017), UALCAN (http://ualcan.path.uab.edu/) (Chandrashekar et al., 2017), Kaplan-Meier plotter (http://kmplot.com/) (Nagy et al., 2021), TISIDB database (http://cis.hku.hk/TISIDB/) (Ru et al., 2019), the Human Disease Methylation Database (http://bio-bigdata.hrbmu.edu.cn/) (Lv et al., 2012), the PrognoScan database (http://dna00.bio.kyutech.ac) (Mizuno et al., 2009), The Linked Omics database (http://www.linkedomics.org/) (Vasaikar et al., 2018). GEPIA database (http://gepia.cancer-pku.cn/) (Tang et al., 2017) and cbioportal (http://www.cbioportal.org/) (Cerami et al., 2012).

### Prediction and Construction the ceRNA Network

We employ the starbase (www.starbase.sysu.edu.cn) (Rhodes et al., 2004), Targetscan (http://www.targetscan.org/) (Li et al., 2017), miRDB (http://mirdb.org) (Chandrashekar et al., 2017) and miRbase (http://microrna.sanger.ac.uk/) (Nagy et al., 2021) to predict the potential miRNAs that able to binds with miRNAs. We also using the starbase (Rhodes et al., 2004) to analysis the expression and prognosis of miRNAs and lncRNAs. The starbase (www.starbase.sysu.edu.cn) (Nagy et al., 2021) and lncRNASNP (http://bioinfo.life.hust.edu.cn/lncRNASNP/) (Lv et al., 2012) was apply to analysis the upstream lncRNAs that bindings with the miRNAs. Furthermore, we using the starbase to analysis the correlation between the LncRNA/miRNA/mRNA.

### Analysis the Protein and Gene Interation Networks

We employed the STRING (www.string-db.org) (Mering, 2003) and GeneMANIA (http://www.genemania.org) (Warde-Farley et al., 2010) perform protein-protein and gene-gene interaction network analysis of SNX20.

### Analysis the Cell Localization and Coding Potential of lncRNA

We employed the lncLocator (http://www.csbio.sjtu.edu.cn/bioinf/lncLocator2/) (Cao et al., 2018) and CPC2 (http://cpc2.cbi.pku.edu.cn) analysis the Cell localization and coding potential of lncRNA.

### Plasmids Construction and Cell Culture

The cDNA for SNX20 was constructed employ pCDH-CMV-MCS-EF1-Puro vector. The NSCLC related cells mainly purchased from The Kunming Institute of Zoology (KIZ) of Chinese Academy of Sciences (CAS). BEAS-2B and NSCLC related cells lines were cultured using the RPMI1640 medium, this medium contain the 10% fetal bovine serum (FBS) and 1% penicillin/streptomycin. The cDNA for SNX20 primer sequences are list follows: SNX20-PCR-F: ATG​GCA​AGT​CCA​GAG​CAC​CCT​G, SNX20-PCR-R: TCA​GGG​TGT​GGC​GTC​AGG​AGC​CGG​AGC​CA.

### Quantitative Real-Time PCR

The qRT-PCR assay was performed as documented (Jiang et al., 2018). For Real-time RT-PCR assay, indicated cells were lysed by RNAiso Plus (Takara Bio, Beijing, China, Cat# 108-95-2). Total RNA was extracted according to the manufacturer’s protocol, and then reverse transcribed using RT reagent Kit (Takara Bio, Beijing, China, Cat# RR047A; TIANGEN Biotech, Beijing, China, Cat# KR211-02). Real-time PCR was performed by FastStart Universal SYBR Green Master Mix (Roche, Cat# 04194194001; TIANGEN Biotech, Beijing, China, Cat# FP411-02) using an Applied Biosystems 7,500 machine. The primers and antibodies used in this study are shown in xx. The primer sequences are list follows SNX20-F: ACC​TGA​CGG​GCA​CTT​AGA​CA, SNX20-R: AGA​GCA​GTT​TGA​CGT​GCT​TCC; β-actin-F: CTTCGCGGGCGACGAT, β-actin-R: CCA​TAG​GAA​TCC​TTC​TGA​CC. The expression quantification was obtained with the 2−ΔΔCt method.

### Cell Proliferation, Colony Formation and Cell Migration Assays

Cell proliferation, colony formation, tumor sphere formation assay was performed as documented (62). Briefly, for cell proliferation assay, indicated cells were plated into 12-well plates at a density of 1.5 × 104, the cell numbers were subsequently counted each day using an automatic cell analyzer countstar (Shanghai Ruiyu Biotech Co., China, IC 1000). For colony formation assay, indicated cells were seeded in 6-well plate with 600 cells per well supplemented with 2 ml cell culture medium, and the cell culture medium was changed every 3 days for 2∼3 weeks. Indicated cells were fixed with 4% PFA and stained with 0.5% crystal violet.

Cell migration assays was performed as documented (62). Briefly, to produce a wound, the monolayer cells in 6-well plate were scraped in a straight line with pipette tips. Plate was then washed with warm PBS to remove detached cells. Photographs of the scratch were taken at indicated time points using Nikon inverted microscope (Ti-S). Gap width was calculated with GraphPad Prism software. For trans-well assay, 2.5−3×104 cells in 100 μL serum free medium were plated in an 8.0 μm, 24-well plate chamber insert (Corning Life Sciences, catalog no. 3422), with medium containing 10% FBS at the bottom of the insert. Cells were incubated for 24 h, and then fixed with 4% paraformaldehyde for 20 min. After washing, cells were stained with 0.5% crystal violet blue. The positively stained cells were examined under the microscope.

### Dual-Luciferase Assay

Briefly, putative binding sites for miR-301a-3p on the 3′-UTR of SNX20 was predicted by starbase dataBase (http://starbase.sysu.edu.cn/). Wild-type and mutant SNX20 (mut- SNX20 or mut- SNX20) fragments were constructed and inserted downstream of the luciferase reporter gene in the reporter plasmid pGL3 plasmid (Promega). HEK-293T cells (2 × 104 cells/well) were seeded in a 24-well plate and co-transfected with 3′-UTR SNX20 construct and miR-301a-3p mimics or miR Ctrl using Lipofectamine 3,000. Both firefly and Renilla luciferase expressions were measured post-transfection using the Dual Luciferase Kit (Promega) according to the manufacturer’s instructions.

### Western Blotting

The Western Blotting and Immunohistochemistry staining assay was performed as documented (Griffiths-Jones, 2006). Briefly, Cell lysates were collected, perform the Western blot, primary antibody overnight incubation and second antibody incubation. Finally, develop using instrument.The detail information of antibodies employ in our study are as follows: SNX20 antibody (SNX20, FNab08087, Rabbit 1:1,000,) and β-actin (Catalog number: 66009-1-Ig, 1:20,000, Proteinch, Shanghai, China).

### Statistical Analysis

The significance of the data between two assays groups was decided by Student’s t-test, *p* < 0.05 (*), *p* < 0.01 (**), and *p* < 0.001 (***), was considered significantly.

## Results

### SNX20 Was Decreased in Human Cancers

**FIGURE 1:**
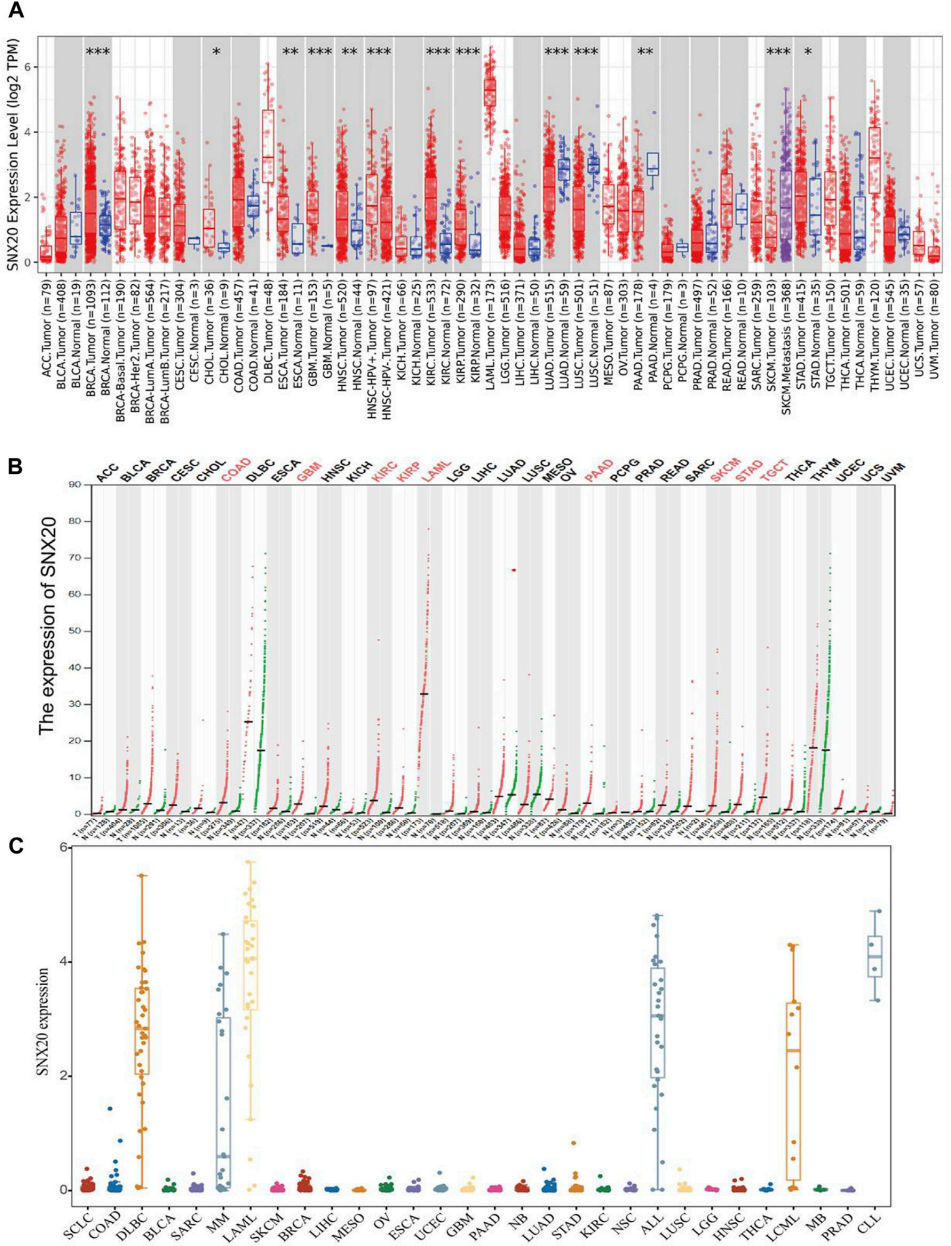
Expression analysis for SNX20 in human cancers. **(A)** SNX20 expression of different tumor types in the TIMER database. **(B)** SNX20 expression in TCGA and GTEx datas. **(C)** SNX20 expression of different tumor cells lines in the CCLE database.

In order to examine the mRNA of SNX20 expression pattern in multifarious cancer, we employed the TIMER tools to analysis the expression of SNX20, the result shown that SNX20 was low expression in LUAD, LUSC, and PAAD, higher expression was observed in BRCA, CHOL, ESCA, GBM, HNSC, KIRC, and KIRP (Figure 1A). To further verify the results, we using the combine the TCGA and GTE databases to figure out the SNX20 expression. As is show in Figure 1B, the SNX20 was significantly up-regulation in COAD, GBM, KIRC, KIRP, LAML, PAAD, SKCM, SARC, and TGCT cancer than match healthy tissue. Besides, we found that the SNX20 was down regulated in NSCLC cells lines observe in CCLE network tools (Figure 1C). Above all, our findings indicated that the SNX20 may plays different roles in the progression of different cancers.

### Correlations of SNX20 Expression With Pathological Stages in Cancers

**FIGURE 2:**
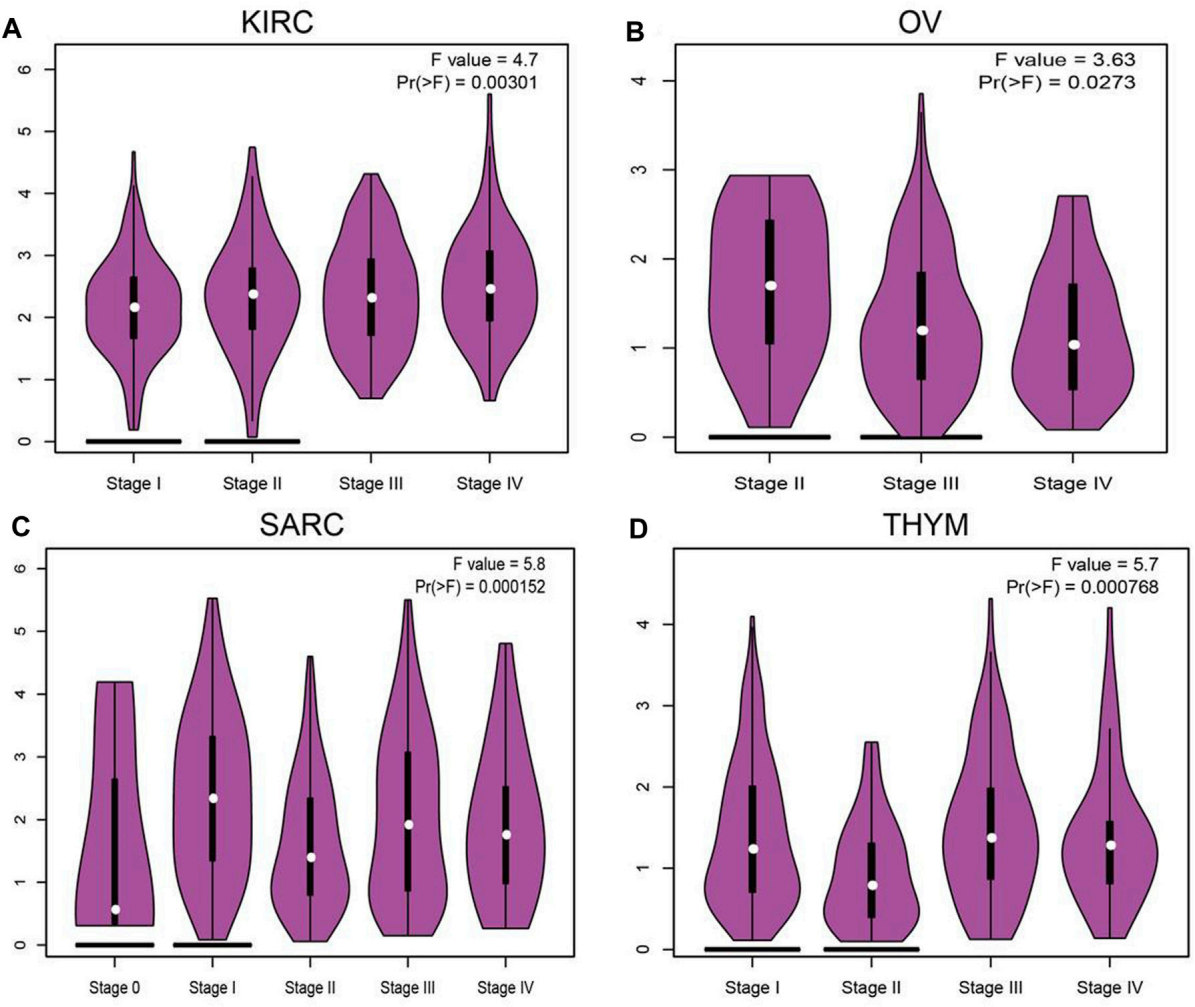
The pathological stage analysis for SNX20 in human cancers. **(A–D)** The pathological stage of SNX20 in KIRC **(A)**, OV **(B)**, SARC **(C)**, and THYM **(D)**.

We employ the GEPIA tools to examine the relationship between the expression of SNX20 and the human cancers pathological stage. Interestingly, we find that the expression of SNX20 was markedly positive with the pathological stage of KIRC, SARC and negative with the pathological stage of OV and THYM (Figures 2A–D). These results suggested that SNX20 plays different roles in different human cancers.

### The Prognostic Values of SNX20 in Human Cancer

**FIGURE 3:**
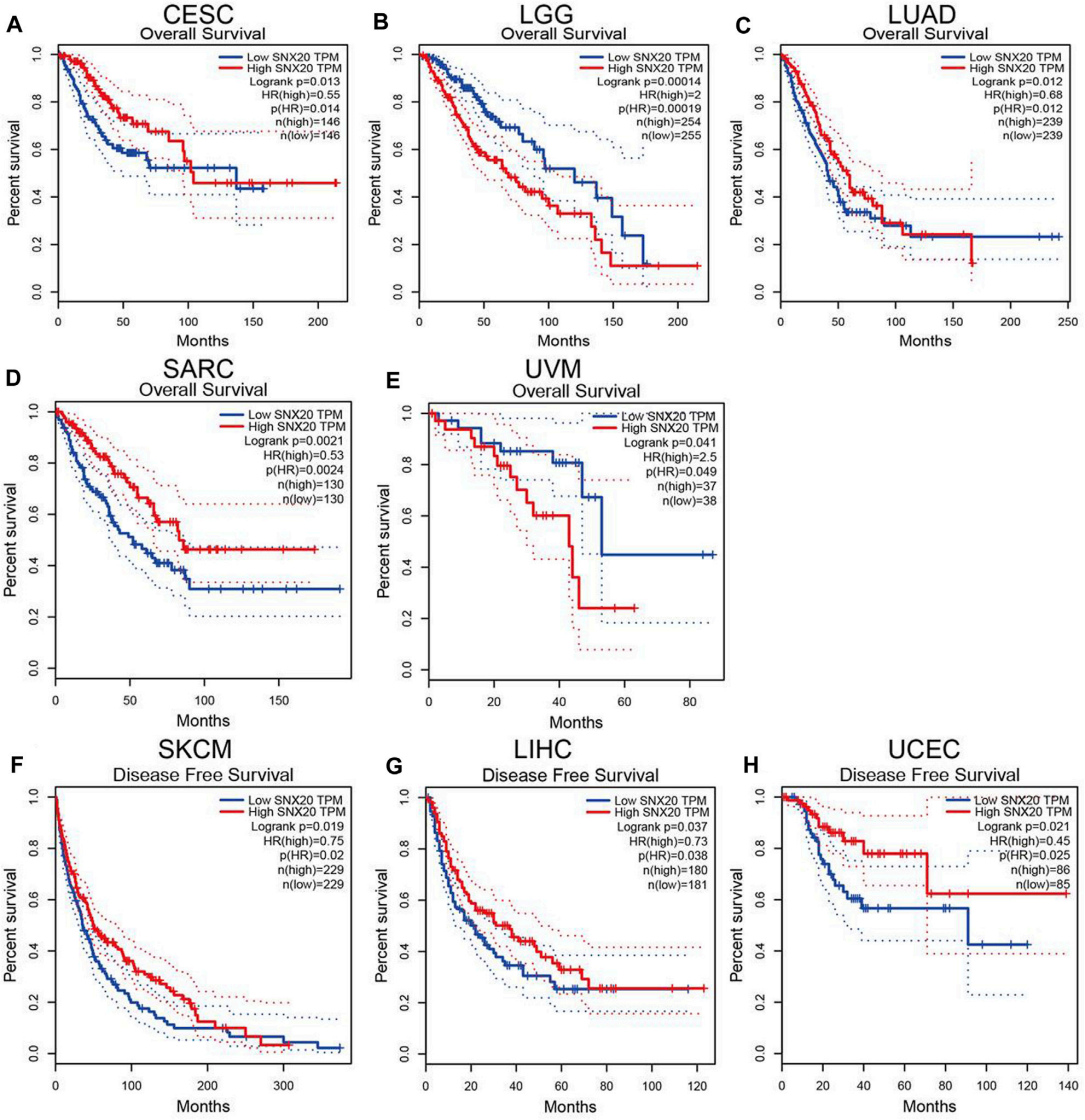
The prognostic values in various cancer subgroups of SNX20. **(A–H)** Prognostic HR of SNX20 in different cancers for OS **(A–E)**, DFS **(F–H)**.

The prognostic value of SNX20 expression in human cancers was analyzed by several databases. In GEPIA, we found that lower SNX20 expression was associated with poorer overall survival (OS) in CESC, LUAD and SARC, the patients with higher SNX20 expression had poor OS in LGG and UVM (Figures 3A–E). Additionally, high expression of SNX20 has a better DFS observed in SKCM, LIHC and UCEC (Figures 3F–H). Lower expression of SNX20 was related to poor DSS in CESC, HNSC, LGG, LUAD, SKCM, UCEC and UVM (Supplementary Figure S1A), and linkage to poor PFS in CESC, HNSC, KIRP and UCEC (Supplementary Figure S1B). The above results proved that SNX20 expression closely related to the prognosis of various cancer types.

### Associations Between SNX20 and Clinical Characteristics of LUAD Patients

**FIGURE 4:**
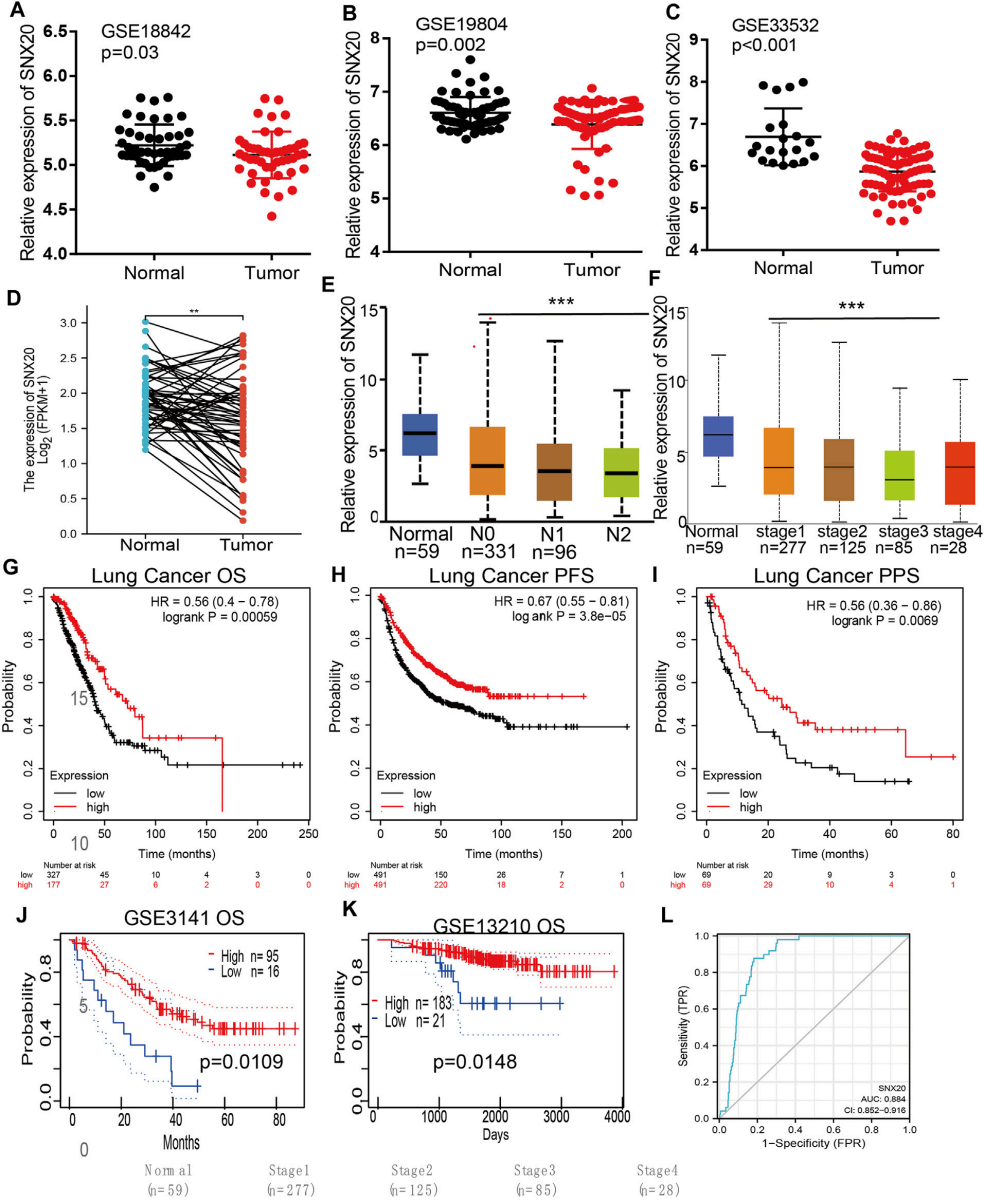
SNX20 was highly expression in NSCLC. **(A–C)** The expression of SNX20 in GEO database. **(D)** The pathological stage analysis for SNX20 in LUAD determined by GEPIA database. **(E)** The expression of SNX20 in 58 pairs of LUAD tissues and adjacent normal tissues. **(F)** The mRNA of SNX20 in patient’s tumor stage by UALCAN database. **(G–I)** The different survival state in LUAD, includings OS, PFS, PPS. **(J–K)** The different survival state in GEO databases, includings OS, RFS. **(L)** ROC curve analyses and AUC values for SNX20 in TCGA LUAD patients.

Considering the significance of SNX20R in cancers, next, we want to exploration the correlation between SNX20 expression and clinical features in LUAD. First, we found that the lower expression was observed in the three GEO datasets (Figures 4A–C). Next, we find that the RNA of SNX20 was significantly lower in LUAD by perform the UALCAN tools analysis ((Figure 4D). In addition, we also find the expression of SNX20 was decreased with the elevation of stage nodal metastasis and tumor stage (Figures 4E,F). Surprisingly, we find higher expression of SNX20 has the better OS, PFS and PPS (Figures 4G–I). Finally, we adopt the GEO dataset to verify above results, the studies shown that elevated the SNX20 expression display the better OS and RFS in NSCLC (Figures 4J,K). ROC curve analysis of SNX20 showed an AUC value of 0.884 in TCGA LUAD patients (Figure 4L). These results indicate that SNX20 has the potential to act as a detection index for the diagnosis of lung cancer with high sensitivity and specificity.

### Analysis the Gene Mutation of SNX20 in NSCLC

**FIGURE 5:**
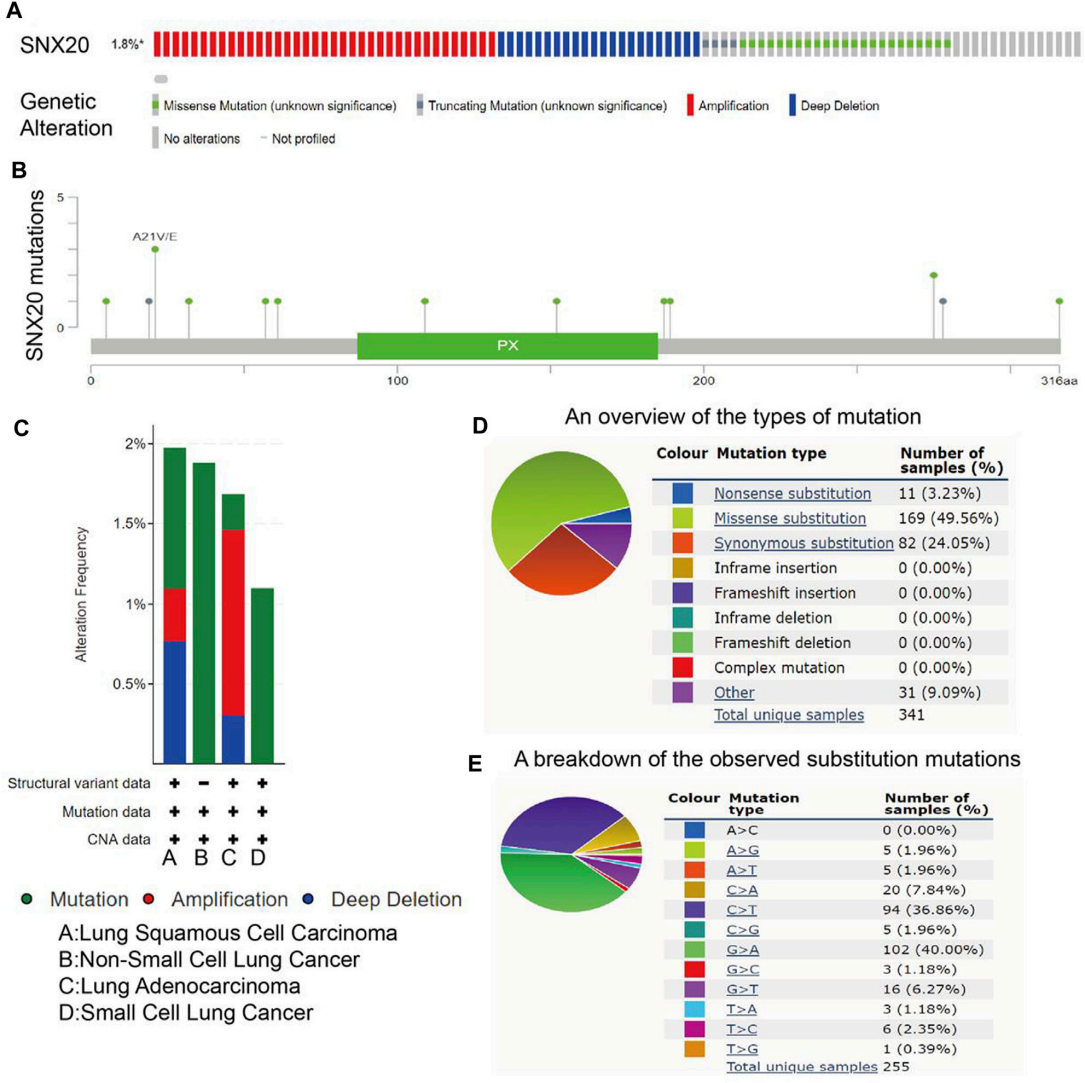
Analysis of the gene mutation of SNX20. **(A–B)** The picture indicated that SNX20 mutations (TCGA) using the cBioportal. **(C)** The picture indicated that gene mutation of SNX20 in different NSCLC histological subtype. **(D–E)** The picture indicated that different mutation types of SNX20 in NSCLC.

For explore the gene mutation information about the SNX20, we employ the cBioportal tools preform comprehensive analysis regard to the SNX20. The result shown that the mutation rate of SNX20 reached 1.8% in NSCLC (Figures 5A,B), the results also display the mutation of SNX20 in different NSCLC molecular Subtypes (Figure 5C), Next, we also examine the mutation type and base mutation in NSCLC, we found that Missense substitution and base G > A reached the highest mutation rate in NSCLC (Figures 5D,E). Overall, these results emphasize the gene mutation of SNX20 may be contribute to the SNX20 low expression in NSCLC.

### Correlations of SNX20 Expression With DNA Methylation

**FIGURE 6:**
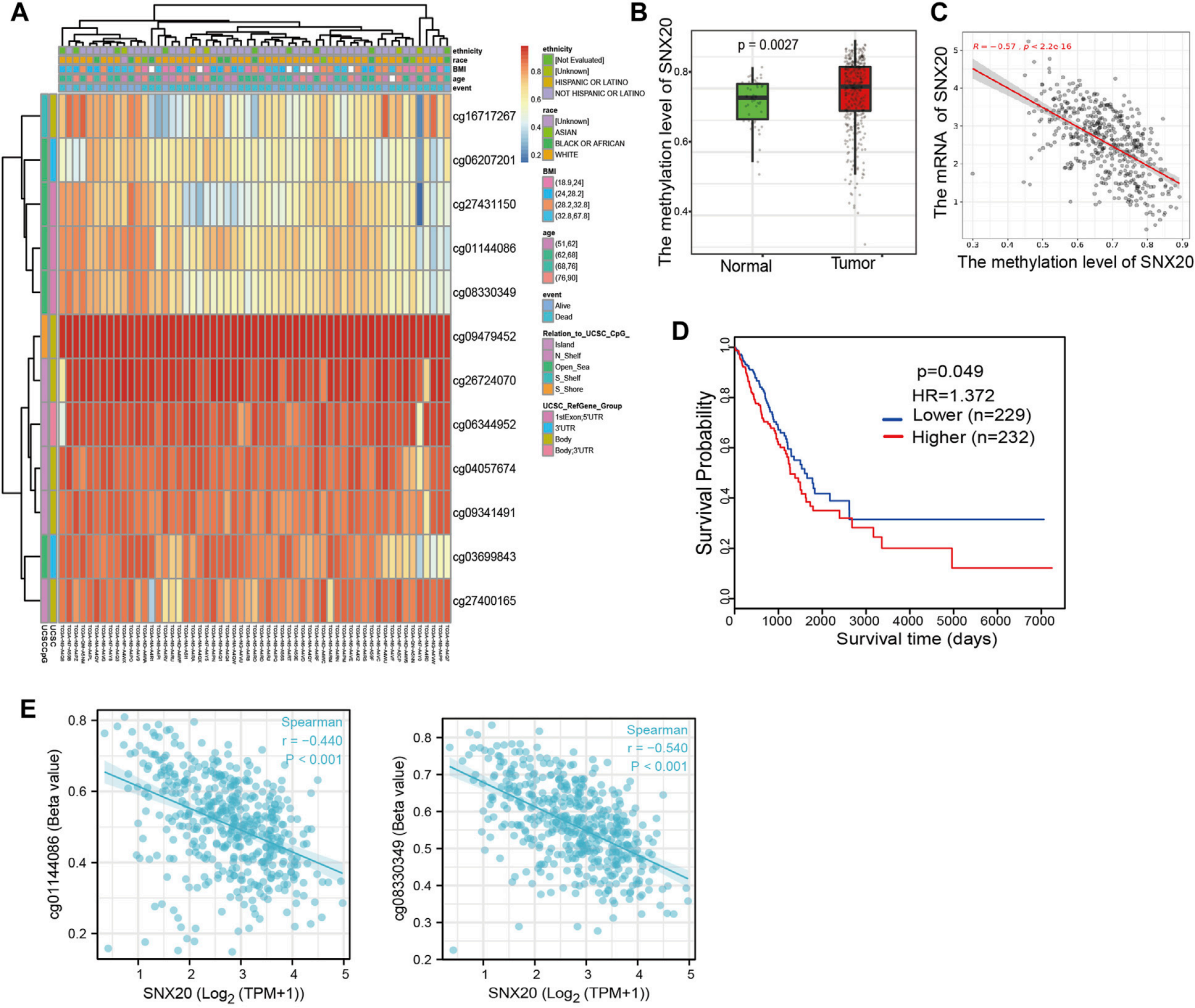
Analysis of the DNA methylation level of SNX20. **(A)** The differential methylation sites linked to SNX20 were show as heatmaps. **(B)** The expression and methylation of SNX20 in LUAD tissues and normal tissues. **(C)** The correlation between the methylation level and mRNA expression of SNX20. **(D)** Survival state of the methylation level of SNX20 in the TCGA LUAD dataset. **(E)** The correlation between the diverse methylation sites and SNX20 expression in LUAD.

As a one of the crucial epigenetic modification, DNA Methylation plays an significant roles in regulation gene expression. In order to explore the molecular mechanism of the SNX20 aberrantly up-regulated in LUAD, we analyzed the promoter methylation levels of SNX20 in LUAD. We found that there are many methylation sites in the promoter region of SNX20, and the differential methylation regions were indicated in the heatmaps (Figure 6A). Importantly, by using the shiny methylation analysis resource tool (SMART) analysis (Tay et al., 2014) we uncovered that the methylation of SNX20 was significantly higher in LUAD cancerous tissues compared to that in normal tissues (Figure 6B). Consistently, we found that the methylation levels on the specific methylation site (cg06207201) within SNX20 promote region negatively correlated with its expression in LUAD (Figure 6C). Furthermore, we showed that the elevated methylation levels on cg06207201 site correlates with worse OS in the TCGA-LUAD cohorts, using the methSurv dataset (Figure 6D). Additionally, we also found that another two methylation sites (cg01144086 and cg08330349) also negatively with the expression of SNX20 in LUAD (Figure 6E). Above all, there results suggested the DNA methylation plays crucial roles in the modulates the expression of SNX20 in LUAD.

### Analysis of the Function of SNX20 in LUAD

**FIGURE 7:**
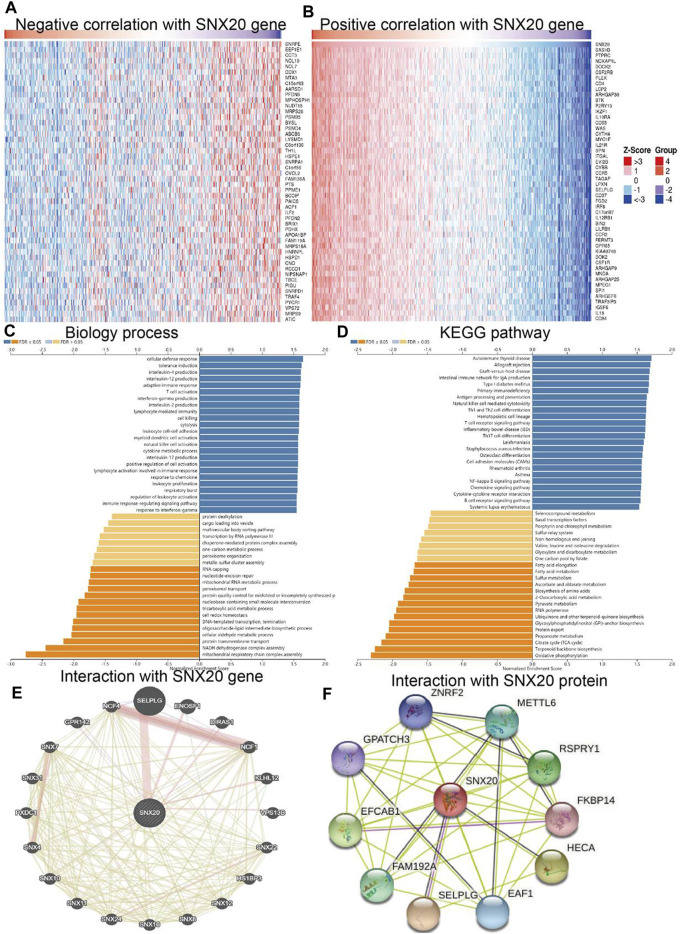
GO and KEGG enrichment analysis for SNX20. **(A–B)** Heat maps of genes positively and negatively correlated with SNX20 (top 50). **(C–D)** Biology process and KEGG pathway analysis of SNX20 by GSEA. **(E)** The gene interaction meshwork of SNX20 was constructed using GeneMania. **(F)** Employ STRING to construction the protein interaction meshwork of SNX20.

To further study the function of SNX20, we employed the Linkedomics to perform a correlation analysis of SNX20. Two heatmaps were constructed to illustrate the genes whose expression was most positively and negatively correlated with that of SNX20 (Figures 7A,B). GO annotation revealed that these genes participate in various immune response, including T cell activation, interferon *γ* production, adaptive immune response, leukocyte proliferation, regulation of defense response to virus by virus, myeloid dendritic cell activation, interleukin-4 production, leukocyte activation involved in inflammatory response, lymphocyte activation involved in immune response, response to chemokine, immune response regulating signaling pathway and regulation of leukocyte activation (Figure 7C). KEGG pathway analysis showed the enrichment in intestinal immune network for IgA production, primary immunodeficiency, hematopoietic cell lineage, T cell receptor signaling pathway, cell adhesion molecules, Th1 and Th2 cell differentiation, Th17 cell differentiation, Natural killer cell medicated cytotoxicity and Fc epsilon RI signaling pathway (Figure 7D). Furthermore, we also employed the GeneMania and STRING databases construction the gene and protein interaction networks of SNX20 in LUAD. The results indicated that the twenty frequently altered genes was correlated with SNX20, including SELPLG, ENOSF1 and NCF (Figure xx), the significantly correlated with the SNX20 protein mainly including ZNRF2, METTL6, FKBP14, HECA and SELPLG (Figures 7E,F).

### Identification of SNX20-Associated Signaling Pathways Using GSEA

**FIGURE 8:**
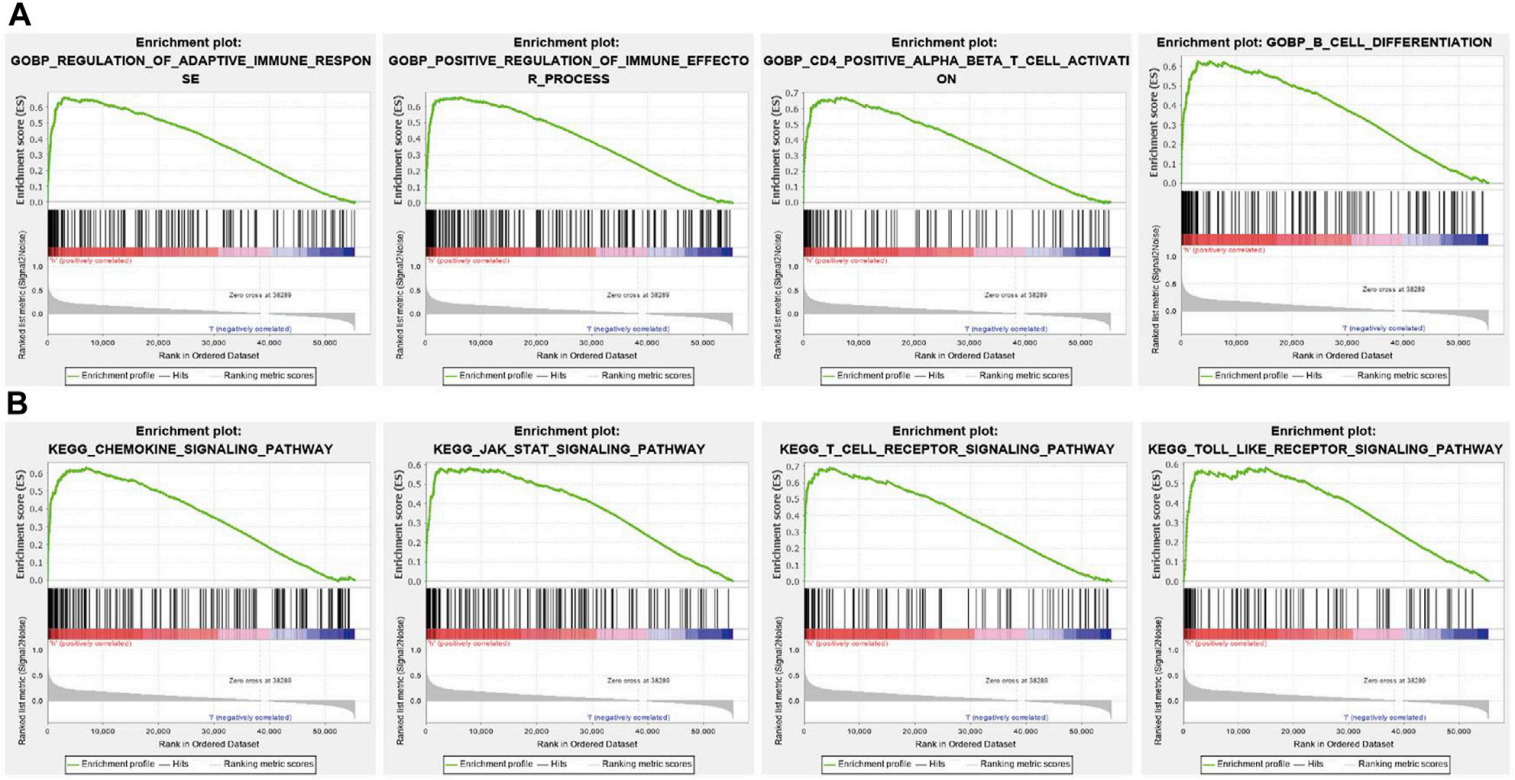
GSEA for SNX20. **(A)** The GO enriched result for SNX20 expression. **(B)** The KEGG pathway enriched result for SNX20 expression.

To further explore the molecular mechanisms affected by SNX20 in Lung adenocarcinoma, we perform the GSEA enriched analysis and find SNX20 mainly participate in the immune related biology processes. GO enriched results indicated that SNX20 were enriched mainly in regulation of adaptive immune response, positive regulation of immune effector process, CD4 positive *β* T cell activation and B cell differentiation (Figure 8A). Similarly, The KEGG enriched results shown that SNX20 were enriched mainly involve in chemokine signaling pathway, JAK STAT signaling pathway, T cell receptor signaling pathway and Toll like receptor signaling pathway (Figure 8B). These results strongly indicated that SNX20 was mainly involved in regulation the immune response of lung adenocarcinoma.

### Analysis the Upstream miRNA of SNX20

**FIGURE 9:**
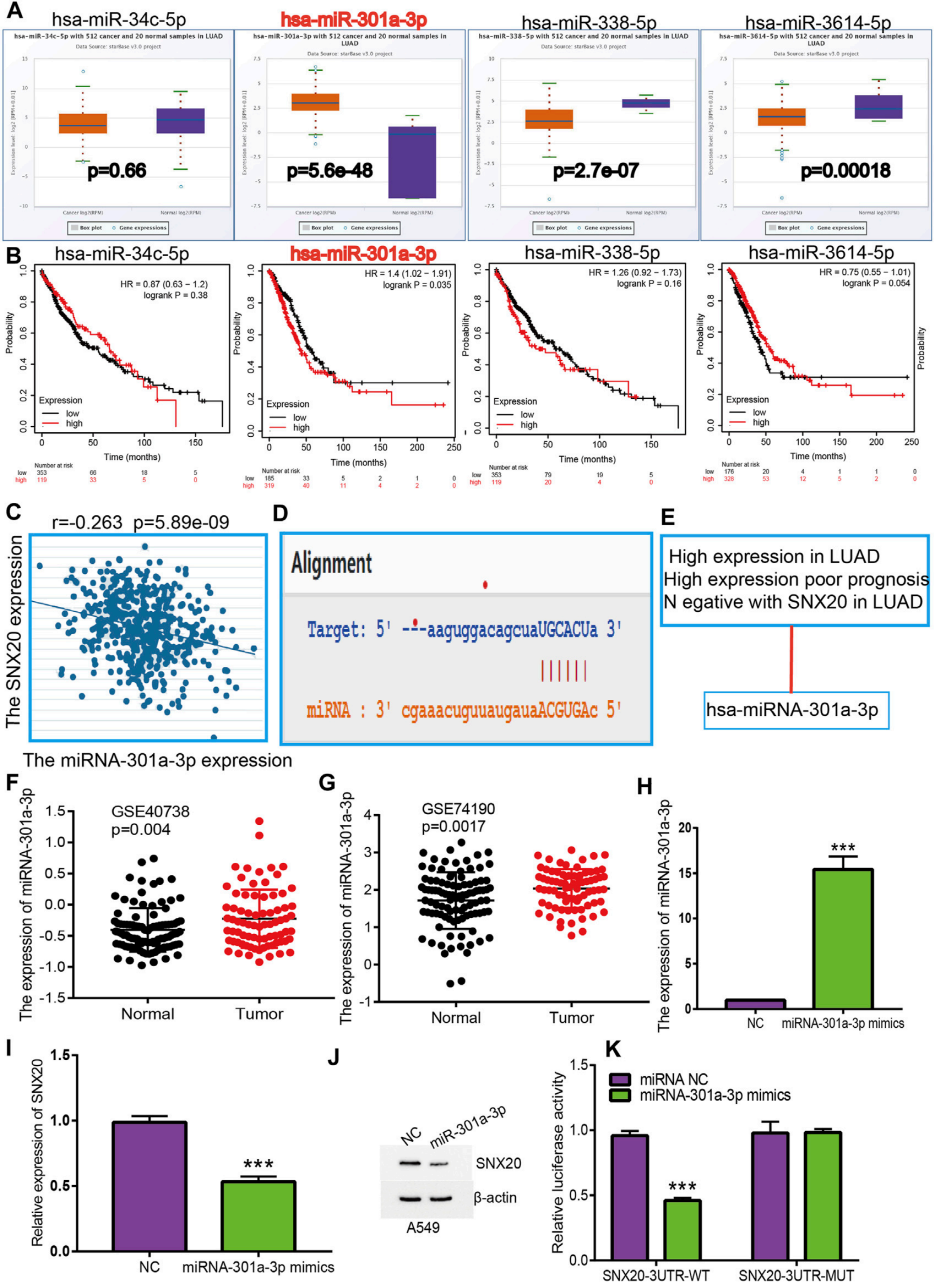
Identification of miRNA-301a-3p could binding the SNX20 in LUAD. **(A)** The expression of miRNA-34c-5p, miRNA-301a-3p, miRNA-338-5p, and miRNA-3614-5p in TCGA-LUAD. **(B)** The prognostic values of miRNA-34c-5p, miRNA-301a-3p, miRNA-338-5p and miRNA-3614-5p in TCGA-LUAD. **(C)** The correlation between expression of miRNA-301a-3p and SNX20 in LUAD. **(D)** Base pairing between miRNA-301a-3p and the SNX20L 3′ UTR predicted by starBase. **(E)** The reasons for obtaining miRNA-301a-3p. **(F)** The expression of miRNA-301a-3p in GSE40738 and control group examine *via* GEO database. **(G)** The expression of miRNA-301a-3p in GSE74190 and control normal samples determined by GEO database. **(H)** The expression of miRNA-301a-3p in A549 cell after transfection miRNA-301a-3p mimics examined by using qRT-PCR assay. **(I)** The expression of SNX20 in A549 cell after transfection miRNA-301a-3p mimics examined by using qRT-PCR assay. **(J)** The protein of SNX20 in A549 cell after transfection miRNA-301a-3p mimics examined by using Western blot assay. **(K)** The relative luciferase activities were analyzed in A549 cell cotransfected with the miR-301a-3p mimics or mimics NC and the SNX20 3′UTR wild-type (WT) or mutant (MUT) luciferase reporter vectors.

To investigate whether some miRNAs regulates the SNX20 expression by miRNA sponge manner, we employed the starbase (Li et al., 2014), Targetscan (Agarwal, 2015), miRDB (Wong and Wang, 2015) and miRbase (Griffiths-Jones, 2006) to predict the potential miRNAs that able to binds with miR-301a-3p. The results find that has-miR-34c-5p, has-miR-301a-3p, has-miR-338-5p, and has-miR-3614-5p may be bind with SNX20. Next, we employed the Starbase and Kmplot databases analysis the expression and prognosis of SNX20 in Lung cancer (Figures 9A,B). According to competing endogenous RNAs hypothesis, the miRNA expression would be negative with the mRNA expression (Tay et al., 2014). Among there miRNAs, only has-miR-301a-3p expression was negatively correlated with SNX20 in LUAD patients (Figure 9C), we also analysis the base pair relationship between the miR-301a-3p and SNX20 (Figure 9D). By perform comprehensive analysis the expression, prognosis and expression correlation, we confirmation the miR-301a-3p may be as aN miRNA sponge for SNX20 (Figure 9E). Furthermore, we adopt the NSCLC GEO dataset to verify the expression of miR-301a-3p in lung cancer, the analysis results shown that miR-301a-3p expression lower in the whole blood and tissue in human lung cancer (Figures 9F,G). To investigate the effects of miR-301a-3p on the expression of target gene, we conducted overexpression analysis. Overexpressing miR-301a-3p significantly reduced both mRNA and protein expressions of SNX20 in A549 cell (Figures 9H–J). The luciferase assay showed that transfection of miR-30a-5p mimics significantly reduced the relative luciferase activity of SNX20-3UTR-WT-treated lung cancer cells, but did not affect that of SNX20-3UTR-MUT-treated lung cancer cells (Figure 9K). All these results confirmed the tumor suppressor roles of miRNA-30a-5p in the lung cancer progression.

### SNX20AR Functions as a Sponge for miR-301a-3p

**FIGURE 10:**
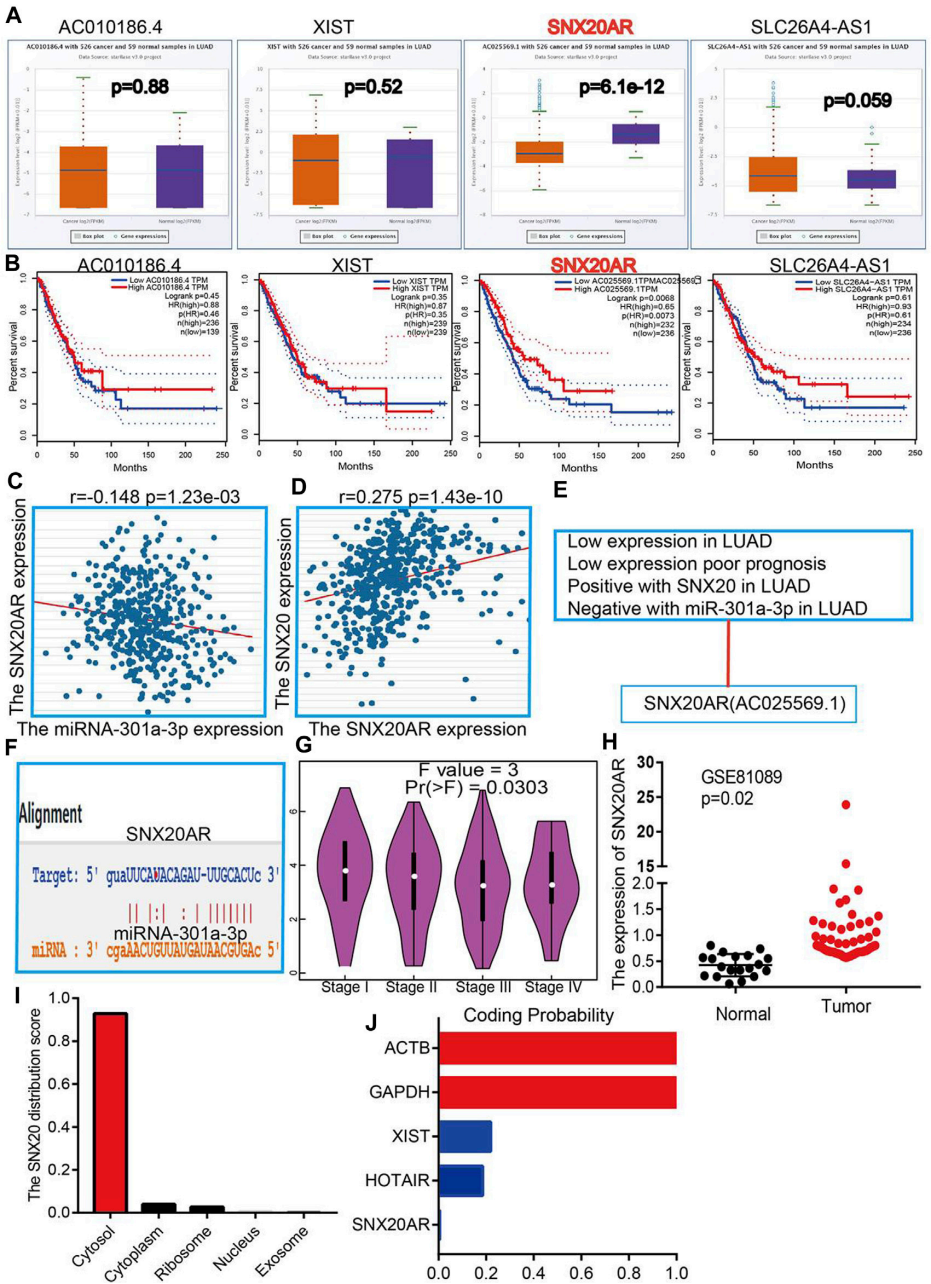
Forecast the Upstream lncRNAs of miRNA-301a-3p. **(A)** The expression of AC010186.4, XIST, SNX20AR and SLC26A4-AS1 in TCGA-LUAD. **(B)** The prognostic values of AC010186.4, XIST, SNX20AR and SLC26A4-AS1 in TCGA-LUAD. **(C)** The correlation between expression of miRNA-301a-3p and SNX20AR in LUAD. **(D)** The correlation between expression of SNX20 and SNX20AR in LUAD **(E)** The reasons for obtaining SNX20A. **(F)** Base pairing between miRNA-301a-3p and the SNX20AR predicted by starBase. **(G)** The pathological stage analysis for SNX20AR in LUAD determined by GEPIA database. **(H)** The expression of SNX20AR in GSE81089 and control normal samples determined by GEO database. **(I–J)** Analysis the Cell localization and coding potential of lncRNA by employed the lncLocator and Coding Potential Calculator.

To explore the upstream lncRNAs that binding with miR-301a-3p, we employed the starbase (Nagy et al., 2021) and lncRNASNP (Gong et al., 2015) analysis the upstream lncRNAs. We find 4 possible lncRNA, including the AC01086.4, XIST, SNX20AR and SLC26A4-AS1. We first examine the expression and prognosis of lncRNAs by employed the starbase and Kmplot (Figures 10A,B). According to competing endogenous RNAs hypothesis, the miRNA expression would be negative with the lncRNA expression, and lncRNA should positive correlation with the mRNA expression (Vasaikar et al., 2018). Owing to the mRNA was decreased expression in LUAD and miRNA was highly expression in LUAD, the lncRNA should be low expression in LUAD. By perform comprehensive analysis the expression, prognosis and expression correlation, we confirmation the SNX20AR may be as an lncRNA sponge for miR-301a-3p. Next, the correlation analysis indicated that the SNX20AR expression not only negatively correlated with miR-301a-3p, but also positively correlated with SNX20 in LUAD (Figures 10C,D).We also analysised the base pair relationship between the miR-301a-3p and SNX20AR (Figure 10F). Finally, we find that the SNX20AR expression was decreased with the cancer stage elevated, and adopt the NSCLC GEO dataset to verify the expression of SNX20AR in lung cancer, the analysis results shown that SNX20AR expression lower in the in human lung cancer (Figures 10G,H). In order to explored the molecular characteristics of SNX20AR, we perform the related analysis by employed the lncLocator (Cao et al., 2018) and Coding Potential Calculator (CPC) (Kong et al., 2007) find that the SNX20AR not only mainly in the cytoplasm, but also no possess protein coding potential (Figures 10I,J). Thus, the data show that SNX20AR may be as a sponge for miR-33a-5p in LUAD.

### SNX20 Expression is Correlated With Immune Infiltration and Immune Checkpoints Related Gene Expression in LUAD

**FIGURE 11:**
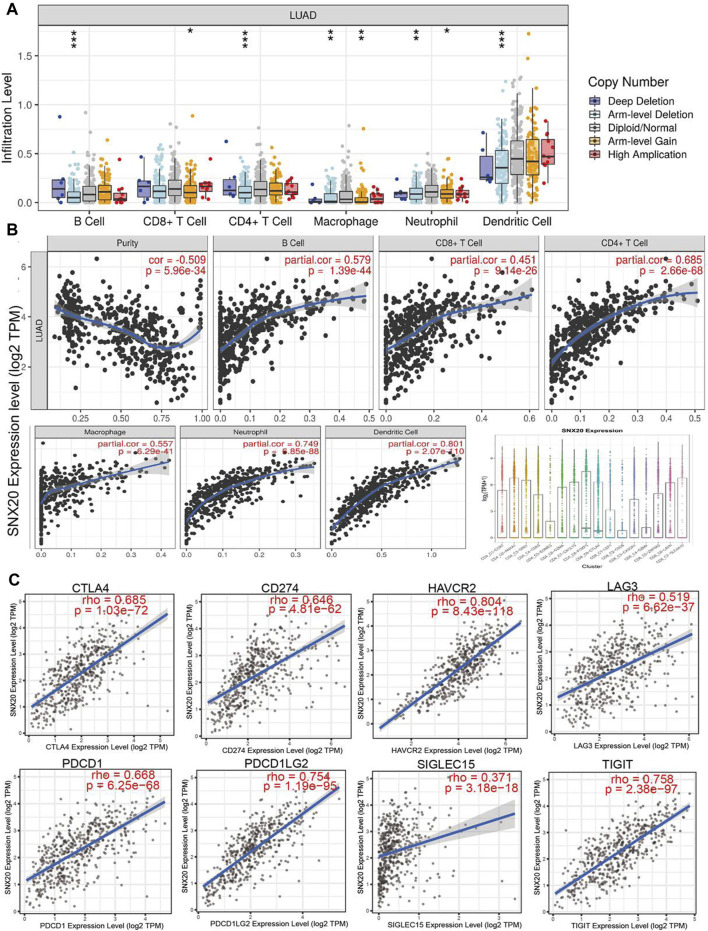
The relationship between immune infiltration and the expression of SNX20 in LUAD. **(A)** The SNX20 gene copy numbers affect the infiltration level of different immune cells in LUAD. **(B)** SNX20 is positively linked to the infiltration of different immune cells employed the TIMER databases. **(C)** SNX20 is positively linked to the immune checkpoints related gene employed the TIMER databases.

To explore the significances of SNX20 in the tumor microenvironment of LUAD. We employed the TIMER to analysis the correlation of SNX20 level with immune infiltration in Lung adenocarcinoma. We find that SNX20 CNV was significantly affect the immune infiltration levels of the immune cells, including the B cell, CD8^+^T cells, CD4^+^T cells, Macrophage, Neutrophil and Dendritic cell (Figure 11A). In addition, we also show that the expression of SNX20 were negatively correlated with tumor purity (r = −0.509, *p* = 5.96e-34) and positively associated with the immune infiltration of B cells (r = 0.579, *p* = 1.39e-44), CD8^+^ T cell (r = 0.451, *p* = 9.14e-26), CD4^+^ T cell (r = 0.685, *p* = 2.66e-28), Macrophage (r = 0.557, *p* = 6.29e-41), Neutrophil (r = 0.801, *p* = 2.07e-110) and Dendritic cell (r = 0.328, *p* = 5.46E-10) in LUAD (Figure 11B). Additionally, we also employed the cox proportional hazard model examine the prognostic value of SNX20 expression and tumor infiltration immune cells in LUAD. The result suggested that B cells (*p* = 0) and CD4^+^ T cells (*p* = 0.014) were significantly associated with clinical prognosis in LUAD (Table 1).

**TABLE 1:** The cox proportional hazard model of SNX20 and different tumor-infiltrating immune cells in LUAD by perform the TIMER databases analysis.

	LUAD				555
	Coef	HR	95%CI_l	95%CI_u	*p*.value
B_cell	−4.888	0.008	0.001	0.098	0
CD8_Tcell	0.466	1.594	0.268	9.488	0.608
CD4_Tcell	3.414	30.401	1.974	468.135	0.014
Macrophage	−0.272	0.762	0.061	9.502	0.833
Neutrophil	0.672	1.958	9.037	104.214	0.74
Dendritic	0.595	1.813	0.414	7.935	0.43
SNX20	−0.382	0.682	0.452	1.029	0.068

Undoubtedly, studies has been demonstrated that immune checkpoints play an crucial roles in the development of cancer immunotherapy (Haanen and Robert, 2015). We employed the TIMER database to exploration the correlation between the SNX20 expression and immune check point related gene. The results suggested that SNX20 was markedly positive with the expression of immune check point related gene, including the CTLA4 (r = 0.685, *p* = 1.03e-72), CD274 (r = 0.646, *p* = 4.81e-62), HAVCR2 (r = 0.804, *p* = 8.43e-118), LAG3 (r = 0.519, *p* = 6.62e-37), PDCD1 (r = 0.668, *p* = 6.25e-68), PDCD1LG2 (r = 0.754, *p* = 1.19e-95), SIGLEC15 (r = 0.371, *p* = 3.18e-18) and TIGIT (r = 0.758, *p* = 2.38e-97) (Figure 11C). Finally, we also employed TISIDB database analysis the relationship between SNX20 level and 28 tumors immune infiltrating cell subtypes and the immune regulator (Table 2). The results suggested that SNX20 was positively with the 28 tumor immune infiltrating cell, immune regulator and MHC molecular (Supplementary Figure S2). These findings indicate that SNX20 plays significant role in cancer immune regulation of LUAD.

**TABLE 2:** The correlation between SNX20 expression and different tumor lymphocyte infiltration in human cancer by perform the TISIDB databases analysis.

	LUAD	
	R	*p*
Activated CD8 T cell (Act _CD8)	0.542	***
Central memory CD8 T cell (Tcm _CD8)	0.498	***
Activated CD4 T cell (Act _CD4)	0.842	***
Central memory CD4T cell (Tcm _CD4)	0.404	***
Effector memory CD4 T cell (Tem _CD4)	0.415	***
T follicular helper cell (Tfh)	0.517	***
Gamma delta T cell (Tgd)	0.846	***
Type 1 T helper cell (Th1)	0.423	***
Type 17 T helper cell (Th17)	0.779	***
Type 2 T helper cell (Th2)	0.485	***
Regulatory T cell (Treg)	0.3	***
Activated B cell (Act _B)	0.782	***
Immature B cell (Imm _B)	0.683	***
Memory B cell (Mem _B)	0.797	***
natural killer cell (NK)	0.464	***
CD56bright natural killer cell (CD56bright)	0.726	***
CD56dim natural killer cell (CD56dim)	0.406	***
Myeloid derived suppressor cell (MDSC)	0.182	***
Natural killer T cell (NKT)	0.853	***
Activated dendtritic cell (Act _DC)	0.716	***
Plasmacytoid dendtritic cell (pDC)	0.62	***
Immature dendtritic cell (iDC)	0.516	***
Macrophage (Macrophage)	0.229	***
Eosinophi (Eosinophil)	0.736	***
Mast (Mast)	0.58	***
Monocyte (Monocyte)	0.46	***
Neutrophil (Neutrophil)	0.425	***

*, **, *** indicate *p*<0.05, *p*<0.01, *p*<0.001, respectively.

### Analysis the Correlation Between the SNX20 Expressions and Immune Cell Type Markers

We estimated the correlation between SNX20 expression and different immune cell gene marker in LUAD by employed the TIMER database. Our results showed that the SNX20 expression was strongly correlation with the different immune markers. For instances, SNX20 expression was strongly correlation with CD8^+^ T markers, CD8A (r = 0.607), CD8B (r = 0.495). The correlation with between the SNX20 expression and immune cells markers as is show in the Table 3. These findings indicated that SNX20 was participate in regulate the tumor immune infiltration in lung adenocarcinoma.

**TABLE 3:** The correlation analysis between SNX20 and different immune cells related gene markers.

Description	Gene markers	LUAD			
		None		Purity	
		Cor	*p*	Cor	p
B cell	CD19	0.523	***	0.394	***
	CD79A	0.482	***	0.354	***
CD8^+^ T cell	CD8A	0.607	***	0.511	***
	CD8B	0.495	***	0.41	***
Dendritic cell	ITGAX	0.756	***	0.702	***
	NRP1	0.314	***	0.298	***
	CD1C	0.553	***	0.491	***
	HLA-DPA1	0.752	***	0.704	***
	HLA-DRA	0.737	***	0.675	***
	HLA-DQB1	0.757	***	0.488	***
	HLA-DPB1	0.571	***	0.702	***
M1 Macrophage	PTGS2	−0.124	*	−0.146	*
	IRF5	0.596	***	0.54	***
	NOS2	0.176	**	0.091	0.56
M2 Macrophage	MS4A4A	0.689	***	0.623	***
	VSIG4	0.658	***	0.611	***
	CD163	0.7	***	0.643	***
Monocyte	CSF1R	0.848	***	0.816	***
	CD86	0.81	***	0.764	***
	KIR2DS4	0.258	***	0.189	***
	KIR3DL3	0.106	***	0.08	***
	KIR3DL2	0.347	***	0.265	***
	KIR3DL1	0.22	***	0.154	***
Natural killer cell	KIR2DL4	0.247	***	0.167	**
	KIR2DL3	0.284	***	0.208	***
	KIR2DL1	0.209	***	0.154	***
Neutrophils	CCR7	0.717	***	0.626	***
	ITGAM	0.785	***	0.753	***
	CEACAM8	0.313	***	0.321	***
T cell (general)	CD3D	0.686	***	0.585	***
	CD3E	0.759	***	0.683	***
	CD2	0.8	***	0.736	***
T cell exhaustion	CTLA4	0.685	***	0.591	***
	LAG3	0.519	***	0.434	***
	HAVCR2	0.804	***	0.755	***
	GZMB	0.428	***	0.314	***
	PDCD1	0.668	***	0.592	***
TAM	CCL2	0.498	***	0.404	***
	IL10	0.623	***	0.535	***
	CD68	0.689	***	0.641	***
Tfh	BCL6	0.144	***	0.148	***
	IL21	0.427	***	0.383	***
Th1	TBX21	0.661	***	0.578	***
	STAT4	0.609	***	0.51	***
	STAT1	0.517	***	0.45	***
	IFNG	0.489	***	0.401	***
Th2	GATA3	0.544	***	0.439	***
	STAT6	0.294	***	0.345	***
	STAT5A	0.785	***	0.739	***
Th17	STAT3	0.216	***	0.252	***
	IL17A	0.256	***	0.189	***
Treg	FOXP3	0.764	***	0.707	***
	CCR8	0.776	***	0.728	***
	STAT5B	0.476	***	0.496	***
	TGFB1	0.577	***	0.52	***

*, **, *** indicate *p*<0.05, *p*<0.01, *p*<0.001, respectively.

### Analysis the Prognosis of SNX20 Based on the Different Immune Cells

**FIGURE 12:**
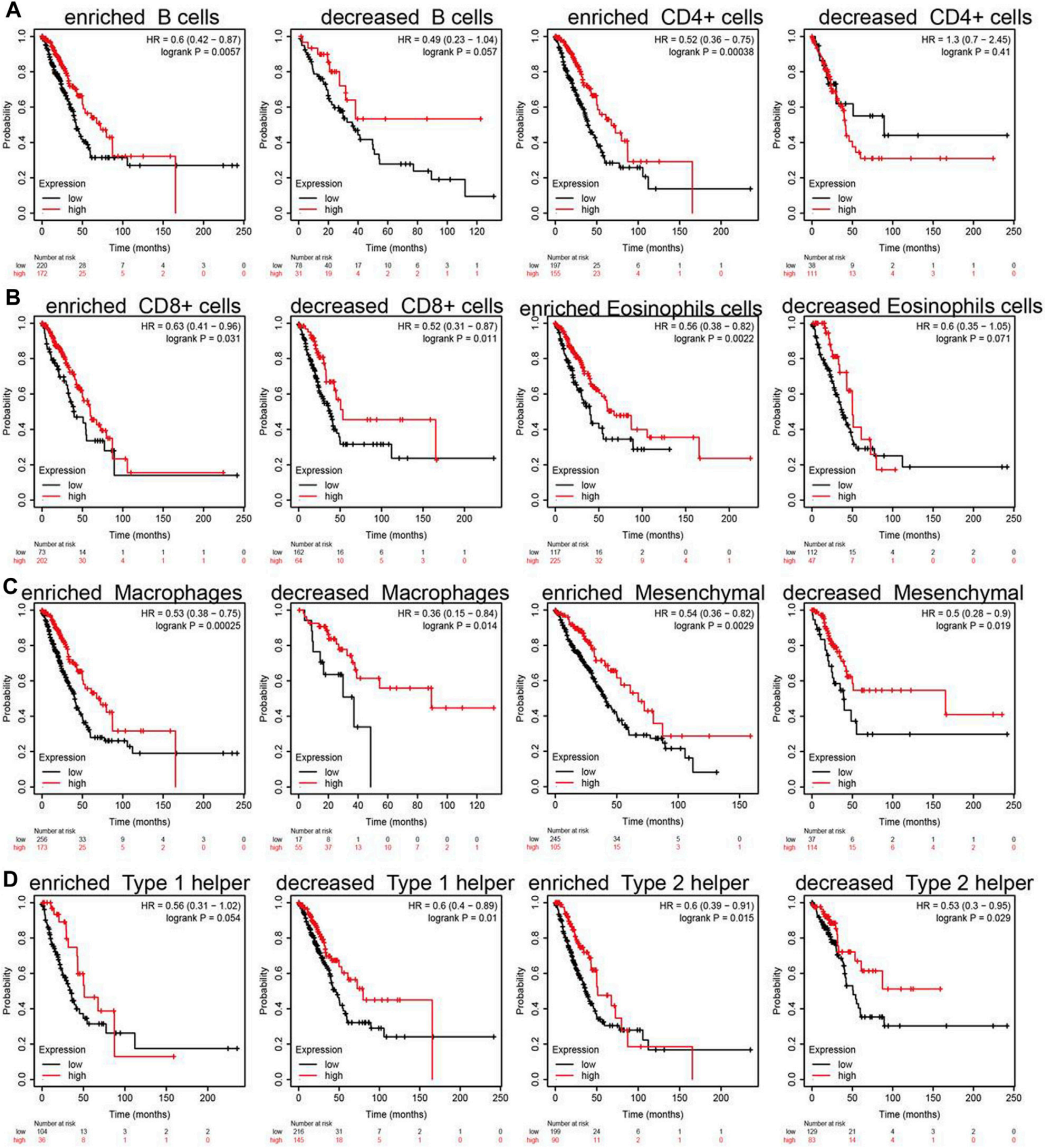
Overall survival curves based on the expression of SNX20 in immune cell subgroups in LUAD. **(A–D)** Correlations between SNX20 expression and OS in different immune cell infiltration group in LUAD patients.

Due to the SNX20 expression can affect the immune infiltration of immune cells. We further explored the expression of SNX20 and the different immune cells infiltration whether influence the prognosis of lung cancer patients. The analysis results suggested that patients with the high expression of SNX20 and enriched the B cells, CD4^+^ cells, CD8^+^ cells, Eosinophils cells, Macrophages cells, Mesenchymal stem cells, Type 1 helper cells and Type 2 helper cells will display a better prognosis. While, The highly expression SNX20 and decreased the B cells, CD4^+^ cells, CD8^+^ cells, Eosinophils cells, Macrophages cells, Mesenchymal stem cells, Type 1 helper cells and Type 2 helper cells will display a poor prognosis (Figures 12A–D). Together, these results suggested that SNX20 expression and different immune cells infiltration would be affect the prognosis of LUAD.

### Over-Expression of SNX20 Suppress Malignant Phenotype of LUAD

**FIGURE 13:**
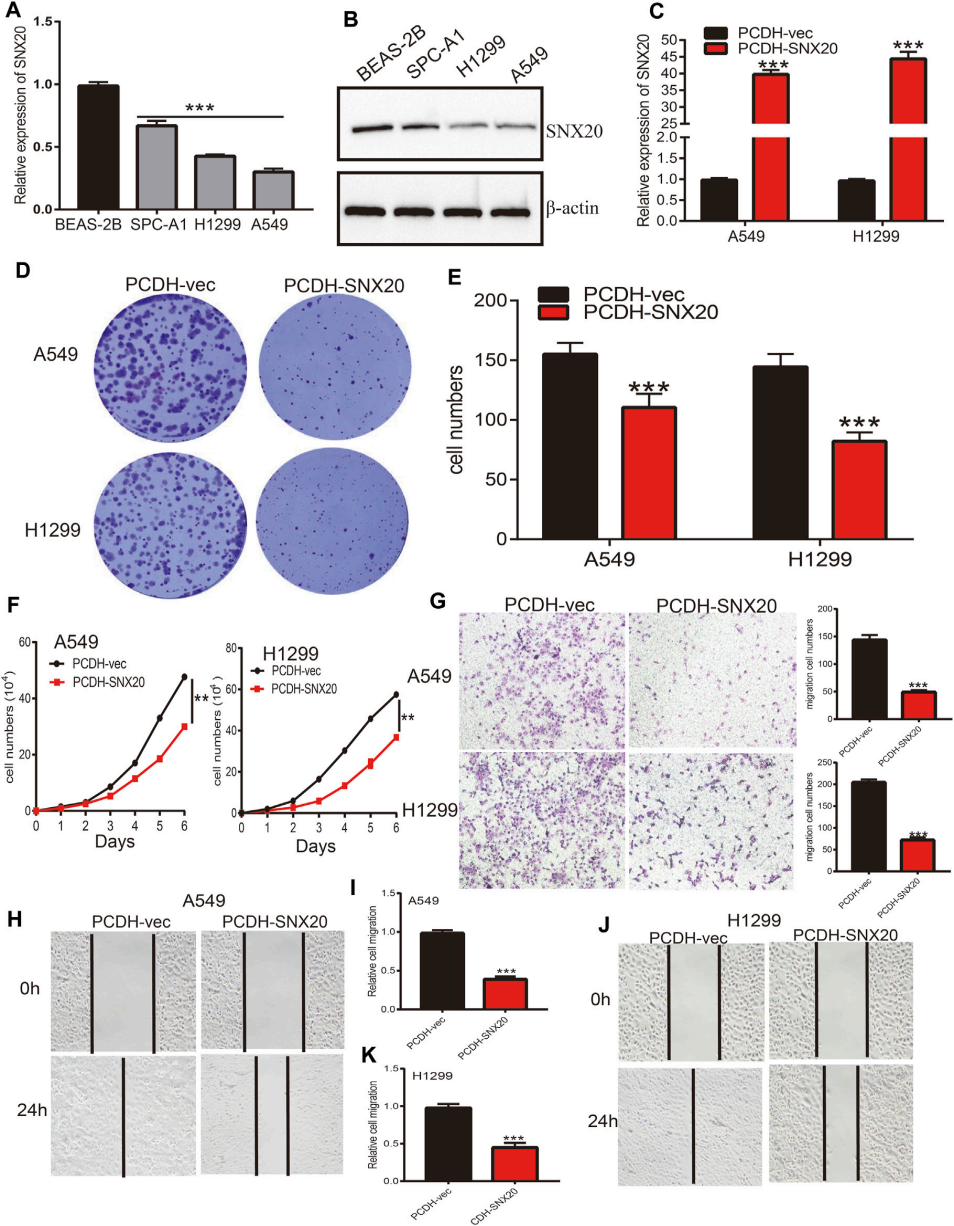
Over-expression of SNX20 on cell proliferation and migration ability of LUAD Cell. **(A)** The mRNA of SNX20 in NSCLC cell lines examined by qPCR assay. **(B)** The protein of SNX20 in NSCLC cell lines examined by Western blot assay. **(C)** Establishment of SNX20 over-expression in NSCLC cells lines and verified by PCR assay. **(D–E)** The colony formation experiment was employed detect over-expression of SNX20 on the growth of NSCLC cells. **(F)** The growth curve experiment was employed detect over-expression of SNX20 on the growth of NSCLC cells. **(G)** The transwell experiment was employed detect over-expression of SNX20 on the migration of NSCLC cells. **(H–K)** The wound healing experiment was employed detect over-expression of SNX20 on the migration of NSCLC cells. **(I)** is the quantification data for **(H)**, **(K)** is the quantification data for **(J)**.

In order to explored the function of SNX20 in LUAD progression. We first examine the SNX20 mRNA expression in NSCLC cells lines. The result show that the mRNA and protein levels of SNX20 were decreased in NSCLC cells, especially in A549 and H1299 cells (Figures 13A,B). Considering the SNX20 was low expression in NSCLC cells, we construction stable overexpression SNX20 cells and using the qRT-PCR assay detection the over-expression efficiency (Figure 13C). Furthermore, we perform the gain of function to examine elevated the SNX20 expression whether affect the proliferation and migration ability of NSCLC cells. The growth curve and clone information experimental result shown that elevated SNX20 was significantly suppressed the cell growth ability of NSCLC cells (Figures 13D–F). Similarly, decreased the migration ability of NSCLC cells was observed in over-expression of SNX20 NSCLC cells (Figures 13G–K). The above findings indicated that SNX20 plays tumor suppressor role in the LUAD progression.

### Analysis the Correlation Between the SNX20 Expression and Drug Sensitivity

**FIGURE 14:**
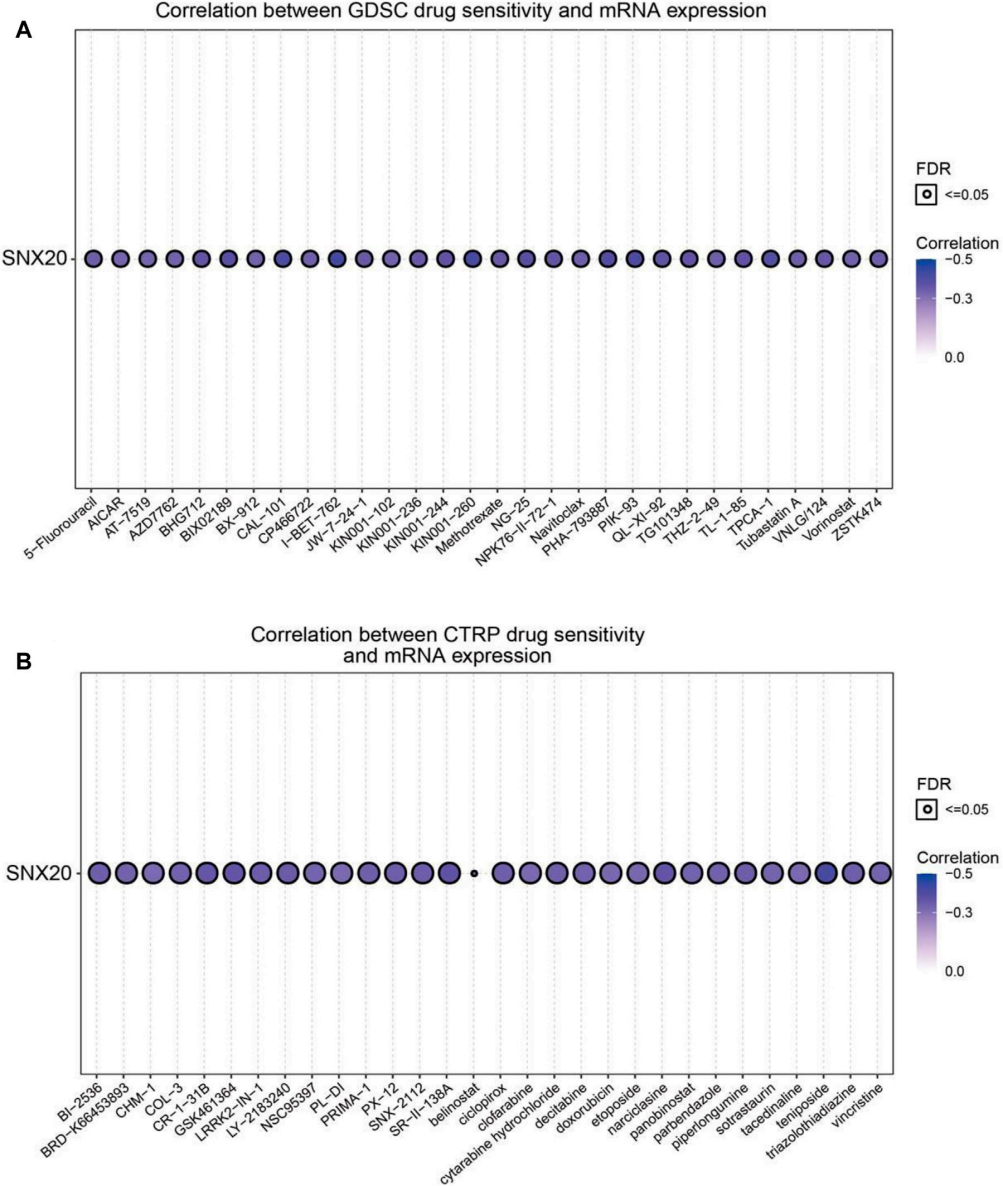
Analysis the correlation between the SNX20 expression and Drug sensitivity in diverse human cancer. **(A)** The correlation between the SNX20 expression and Drug sensitivity in diverse human cancer analysis by employed GDSC database. **(B)** The correlation between the SNX20 expression and Drug sensitivity in diverse human cancer analysis by employed CTRP database.

Above results suggested that SNX20 may plays oncogene roles in the cancer progression, so we next explored the correlation between SNX20 expression and different drug sensitivity in different cancer cell lines from the GDSC and CTRP database. The result indicated that SNX20 expression was negatively correlated with drug sensitivity of I-BET-762, KIN001-260, CAL-101, PIK-93, PHA-793887, BIX02189, TPCA-1, NG-25, QL-XI-92, KIN001-244, TG101348, TL-1-85, BHG712 and NPK76-II-72-1in GDSC database (r < −0.36, Figure 14A). In CTRP database, we observed SNX20 expression was negatively correlated with the drug sensitivity of teniposide, BRD-K30748066, SR-II-138A, Narciclasine, GSK461364, CR-1-31B, LY-2183240, LRRK2-IN-1, SNX-2112, piperlongumine, ciclopirox, PX-12 and triazolothiadiazine (r < −0.35, Figure 14B). In summary, these results demonstrate that SNX20 was significantly related to diverse drug sensitivity in the different cancer cell lines.

## Discussion

Studies have shown that SNX20 plays an crucial roles in endosome-associated scaffolds (Clairfeuille et al., 2015). However, the functions and molecular of SNX20 in the progression of LUAD was remain puzzled. In this study, we analysis the SNX20 expression in human cancers, the results shown that SNX20 was elevated in the nine cancers. However, SNX20 was low expression in LUAD and LUSC, The GEO cohort also find that SNX20 was low expressed in LUAD, using the TCGA LUAD and GEO dataset analysis found that elevated the SNX20 expression display a better prognosis, SNX20 expression was associated with the tumor stage and lymph node metastasis (Figure 4). Fan et al. (2020) found that high SNX20 expression has a better overall survival than patients with low expression SNX20 group. However, no study focus on the functions analysis of the SNX20 co-expression genes. In our study, we employed the LinkedOmics tools to examine the SNX20 co-expression in LUAD. Next, we perform the GO and KEGG enriched analysis the SNX20 co-expression genes, the GO term mainly involve in the T cell activation, adaptive immune response, leukocyte proliferation, regulation of defense response to virus by virus, myeloid dendritic cell activation, interleukin-4 production, leukocyte activation involved in inflammatory response, lymphocyte activation involved in immune response, response to chemokine, immune response regulating signaling pathway and regulation of leukocyte activation (Figure 7C). The KEGG pathway mainly involve in the intestinal immune network for IgA production, primary immunodeficiency, hematopoietic cell lineage, T cell receptor signaling pathway, cell adhesion molecules, Th1 and Th2 cell differentiation, Th17 cell differentiation, Natural killer cell medicated cytotoxicity and Fc epsilon RI signaling pathway (Figure 7D). Finally, our GSEA results shown that SNX20 expression was participate in the adaptive immune response, positive regulation of immune effector process, CD4 positive *β* T cell activation and B cell differentiation (Figure 8A). Similarly, The KEGG enriched results shown that SNX20 were enriched mainly involve in chemokine signaling pathway, JAK STAT signaling pathway, T cell receptor signaling pathway and Toll like receptor signaling pathway (Figure 8B). These results strongly indicated that SNX20 was mainly involved in regulation the immune response of lung adenocarcinoma. With regard to the function of SNX20 in LUAD, analysis the protein-protein interaction network shown that SNX20 was significantly correlated with the NCF1, KLHL12, ENOSF1 and HS1BP3. These proteins all play crucial roles in the regulation of immune microenvironment. It has been shown that depletion of NCF1 able to inhibits the cell growth of melanoma (Kelkka et al., 2013). As a one of E3 ligase, KLHL12 play crucial role in the regulation of wnt and Planar Cell Polarity Signaling (Shami Shah et al., 2019). Søreng (2017) found that HS1BP3 was negative regulate the autophagy process *via* inhibits of PLD1 activity and localization. Yang et al. (2015) suggested that ENOSF1 could be as potential serum biomarkers for gastric cancer. The above results indicated that SNX20 may not only involve in cell proliferation in LUAD, but also have important functions in the regulation of immune microenvironment.

To explore the correlation between SNX20 and immune infiltration in LUAD, we employed the TIMER database to examine the immune infiltration related analysis, the results shown that SNX20 CNV was significantly affect the immune infiltration levels of the immune cells, including the B cell, CD8^+^T cells, CD4^+^T cells, Macrophage, Neutrophil and Dendritic cell (Figure 11A). In addition, we also find that the expression of SNX20 were negatively correlated with tumor purity and positively associated with the immune infiltration of B cells (r = 0.579, *p* = 1.39e-44), CD8^+^ T cell (r = 0.451, *p* = 9.14e-26), CD4^+^ T cell (r = 0.685, *p* = 2.66e-28), Macrophage (r = 0.557, *p* = 6.29e-41), Neutrophil (r = 0.801, *p* = 2.07e-110) and Dendritic cell (r = 0.328, *p* = 5.46E-10) in LUAD (Figure 11B). Additionally, our study suggested that SNX20 was markedly positive with the expression of immune check point related gene, including the CTLA4 (r = 0.685, *p* = 1.03e-72), CD274 (r = 0.646, *p* = 4.81e-62), HAVCR2 (r = 0.804, *p* = 8.43e-118), LAG3 (r = 0.519, *p* = 6.62e-37), PDCD1 (r = 0.668, *p* = 6.25e-68), PDCD1LG2 (r = 0.754, *p* = 1.19e-95), SIGLEC15 (r = 0.371, *p* = 3.18e-18) and TIGIT (r = 0.758, *p* = 2.38e-97) (Figure 11C). These findings indicate that SNX20 plays significant role in cancer immune regulation of LUAD.

Perform the TIMER tools analysis shown that SNX20 expression was markedly correlation with the varies immune cells gene markers, including the B cells (CD19), CD8^+^ T Cell (CD8A, CD8B), dendritic cell (ITGAX, NRP1, CD1C, HLA-DAP1, HLA-DRA, HLA-DQB1, HLA-DPB1), M1 Macrophage cells (PTGS2, IRF5, NOS2) M2 Macrophage cells (MS4A4A, CD163), Monocyte cells (CSF1R, CD86, KIR2DS4, KIR3DL3, KIR3DL2, KIR3DL1), Natural killer cells (KIR2DL4,KIR2DL3, KIR2DL1), Neutrophils cells (CCR7, ITGAM, CEACAM8), T cells (CD3D, CD3E, CD2), T cell exhaustion cells (CTLA4, LAG3, HAVCR2, GZMB, PDCD1), TAM cells (CCL2, IL10, CD68), Tfh cells (BCL6, IL21), Th1 cells (TBX21, STAT4, STAT1, IFNG), Th2 cells (GATA3, STAT6, STAT5A), Th17 cells (STAT3, IL17A), Treg cells (FOXP3, CCR8, STAT5B, TGFB1). These studies indicated that SNX20 plays crucial role in the regulation immune infiltrating cells of LUAD (Table 2). We also that SNX20 expression and different immune cells infiltration would be affect the prognosis of LUAD.

We also determined the upstream regulation mechanism of SNX20 in the progression of LUAD. As the important content of epigenetic modification, non-coding RNAs was shown plays significant roles in the regulation gene expression. LncRNA was reported that as an miRNA sponge to competitively binding miRNA and result in change the expression of downstream target genes. For example, the PDL1 related lncRNA was shown that through elevated the c-Myc expression and participate in the progression of lung adenocarcinoma (Qu et al., 2021). Chen et al. (2020) found that LINC00173.v1 *via* inhibits the miR-511-5p to upregulation of VEGFA expression, result in boost the vascular endothelial cells growth and migration in lung squamous cell carcinoma. Yan et.al observed that lncRNA JPX *via* restrain the expression of miR-33a-5p and subsequently up-regulated the Twist1 expression, result in activating Wnt/β-catenin signaling to involve in the progression of lung cancer (Pan et al., 2020). In this study, we first predicted and comprehensive analysis the upstream miRNAs of SNX20 in LUAD. Combine with The 4 databases predicted 4 potential upstream miRNAs, while only miR-301a-3p was highly expression in LUAD and it’s high expression was negative associated with SNX20 in LUAD. Next, we further examine the upstream lncRNAs of miR-301a-3p, by perform the expression and correlation analysis, only SNX20 meet the established conditions. The SNX20AR was significantly decreased in LUAD and it’s low expression was associated with the poor prognosis. Moreover, we find SNX20AR expression was not only markedly positive with the SNX20, but also negative with the miRNA-301a-3p expression in LUAD. Above all, although the SNX20 related ceRNA network was analysed *via* bioinformatics analysis, the accurate and credible assays are needed to verify the proposed hypothesis. We also found that over-expression of SNX20 was significantly inhibits the cell proliferation and migration of NSCLC cells.

**FIGURE 15:**
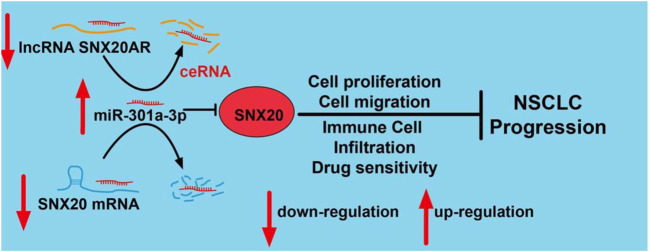
Schematic diagram of the molecular mechanism of SNX20AR/miRNA-301a-3p/SNX20 Axis Associated With Cell Proliferation and Immune Infiltration In Lung Adenocarcinoma.

This work not only deep the understanding of the potential mechanism of lung cancer progression, but also raised our awareness about the tumor-immune microenvironment of lung cancer. Moreover, we revealed that SNX20 was down-regulation in LUAD. The upstream complex molecular regulation mechanism of SNX20 was revealed by us, that is SNX20AR/miRNA-301a-3p (Figure 15). Finally, our work demonstrates that SNX20 was further implicated in alteration of the tumor microenvironment. Over-expression of SNX20 inhibits cell proliferation and cell migration in NSCLC cells. These findings suggested that SNX20 may be plays tumor suppressor roles in the progression of lung cancer and represent an effective target of immunotherapy in the LUAD.

## Data Availability

The original contributions presented in the study are included in the article/Supplementary Material, further inquiries can be directed to the corresponding author.

## Glossary

ACC: Adrenocortical carcinoma
BLCA: Bladder Urothelial Carcinoma
BRCA: Breast invasive carcinoma
CESC: Cervical squamous cell carcinoma and endocervical adenocarcinoma
CHOL: Cholangiocarcinoma
COAD: Colon adenocarcinoma
DLBC: Lymphoid Neoplasm Diffuse Large B-cell Lymphoma
ESCA: Esophageal carcinoma
GBM: Glioblastoma multiforme
HNSC: Head and Neck squamous cell carcinoma
KICH: Kidney Chromophobe
KIRC: Kidney renal clear cell carcinoma
KIRP: Kidney renal papillary cell carcinoma
LAML: Acute Myeloid Leukemia
LGG: Brain Lower Grade Glioma
LIHC: Liver hepatocellular carcinoma
LUAD: Lung adenocarcinoma
LUSC: Lung squamous cell carcinoma
MESO: Mesothelioma
OV: Ovarian serous cystadenocarcinoma
PAAD: Pancreatic adenocarcinoma
PCPG: Pheochromocytoma and Paraganglioma
PRAD: Prostate adenocarcinoma
READ: Rectum adenocarcinoma
SARC: Sarcoma
SKCM: Skin Cutaneous Melanoma
STAD: Stomach adenocarcinoma
TGCT: Testicular Germ Cell Tumors
THCA: Thyroid carcinoma
THYM: Thymoma
UCEC: Uterine Corpus Endometrial Carcinoma
UCS: Uterine Carcinosarcoma
UVM: Uveal Melanoma
