# Supplementary material for: SNX20AR/MiRNA-301a-3p/SNX20 Axis Associated With Cell Proliferation and Immune Infiltration in Lung Adenocarcinoma
Source: Front Mol Biosci. 2021 Sep 17;8:744363. doi: 10.3389/fmolb.2021.744363 (PMC8484765; doi:10.3389/fmolb.2021.744363)
Supplement: Supplementary file 10 [file DataSheet1.docx]

Supplementary Material

1. Supplementary Figures

1.1 Supplementary Figure 1


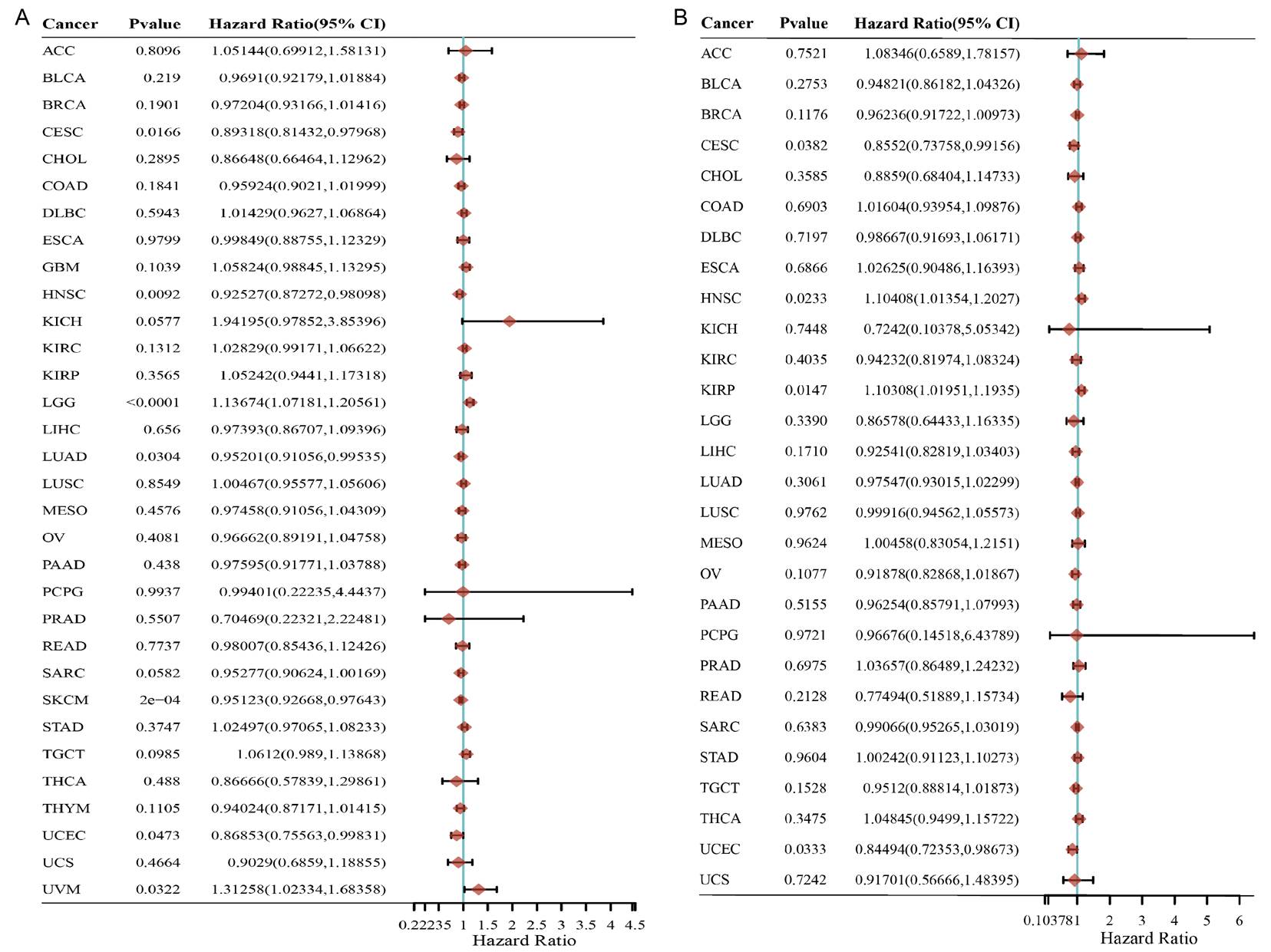


**Supplementary FIGURE 1 ⎜** Association between the SNX20 expression and the DSS and PFS of cancer patients. (A) A forest plot of hazard ratios shown that the DSS of SNX20 in 33 types of tumors. (B) A forest plot of hazard ratios shown that the PFS of SNX20 in 33 types of tumors.

1.2 Supplementary Figure 2


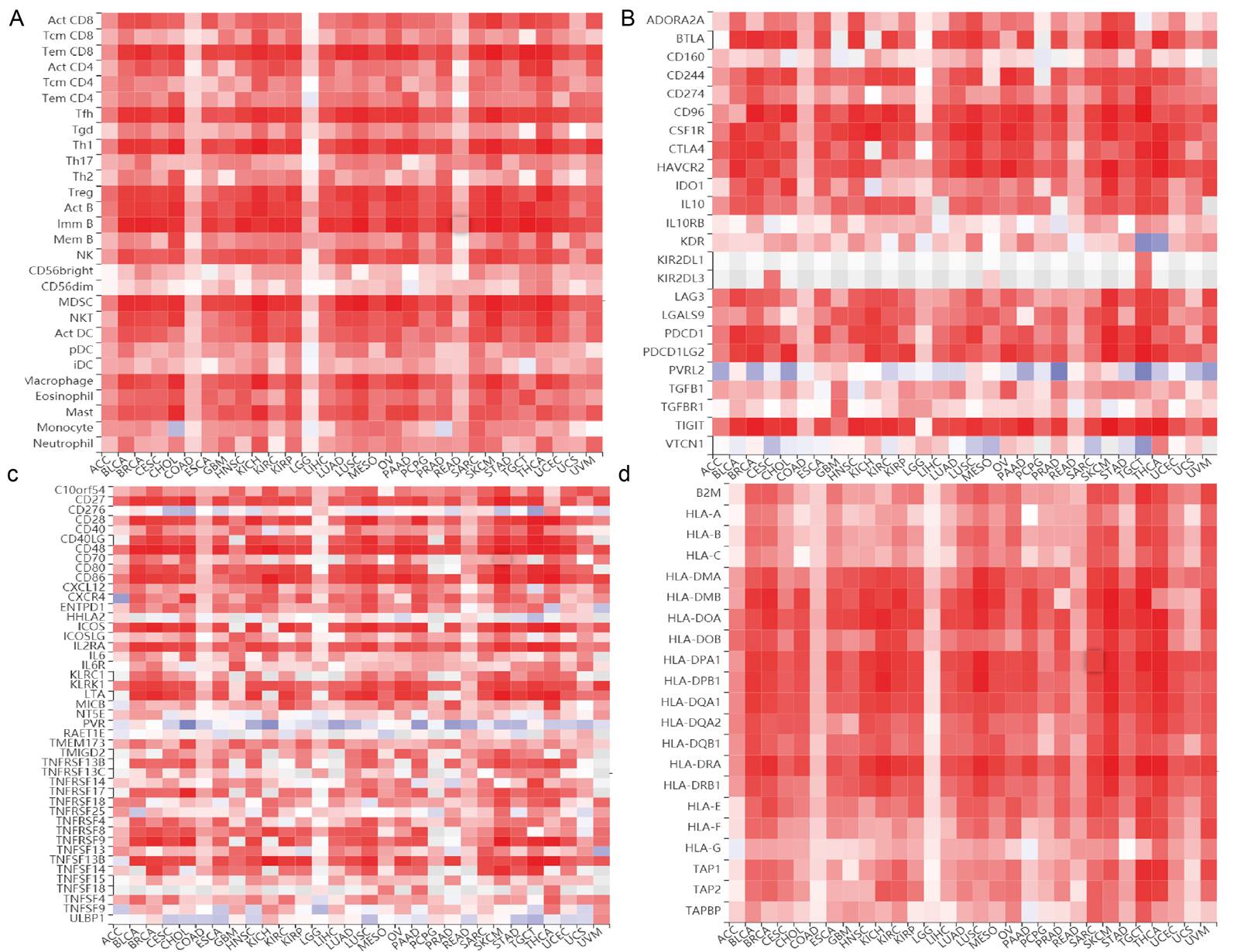


**Supplementary FIGURE 2 ⎜** The relationship between SNX20 expression and TILs across multiple cancer types.

(A) The correlations between SNX20 expression and tumor-infiltrating lymphocytes (TILs) in multiple cancer types.(B) The correlations between SNX20 expression and immunoinhibitor s in multiple cancer types.(C) The correlations between SNX20 expression and immunostimulator s in multiple cancer types.(D) The correlations between SNX20 expression and MHC molecule in multiple cancer types.
